# Silver Nanoparticles in Therapeutics and Beyond: A Review of Mechanism Insights and Applications

**DOI:** 10.3390/nano14201618

**Published:** 2024-10-10

**Authors:** Furkan Eker, Hatice Duman, Emir Akdaşçi, Anna Maria Witkowska, Mikhael Bechelany, Sercan Karav

**Affiliations:** 1 Department of Molecular Biology and Genetics, Çanakkale Onsekiz Mart University, Çanakkale 17100, Türkiye; furkan.eker@stu.comu.edu.tr (F.E.); hatice.duman@comu.edu.tr (H.D.); emirakdasci@gmail.com (E.A.); 2 Department of Food Biotechnology, Medical University of Bialystok, 15-089 Bialystok, Poland; anna.witkowska@umb.edu.pl; 3 Institut Européen des Membranes (IEM), UMR 5635, University Montpellier, ENSCM, CNRS, F-34095 Montpellier, France; 4 Functional Materials Group, Gulf University for Science and Technology (GUST), Masjid Al Aqsa Street, Mubarak Al-Abdullah 32093, Kuwait

**Keywords:** silver nanoparticles, biomedical applications, toxicity, antimicrobial and anticancer activity, bone healing and wound repair activity, bioimaging and biosensor, diabetes, dental applications

## Abstract

Silver nanoparticles (NPs) have become highly promising agents in the field of biomedical science, offering wide therapeutic potential due to their unique physicochemical properties. The unique characteristics of silver NPs, such as their higher surface-area-to-volume ratio, make them ideal for a variety of biological applications. They are easily processed thanks to their large surface area, strong surface plasmon resonance (SPR), stable nature, and multifunctionality. With an emphasis on the mechanisms of action, efficacy, and prospective advantages of silver NPs, this review attempts to give a thorough overview of the numerous biological applications of these particles. The utilization of silver NPs in diagnostics, such as bioimaging and biosensing, as well as their functions in therapeutic interventions such as antimicrobial therapies, cancer therapy, diabetes treatment, bone repair, and wound healing, are investigated. The underlying processes by which silver NPs exercise their effects, such as oxidative stress induction, apoptosis, and microbial cell membrane rupture, are explored. Furthermore, toxicological concerns and regulatory issues are discussed, as well as the present difficulties and restrictions related to the application of silver NPs in medicine.

## 1. Introduction

Silver ions have a long historical background in antimicrobial applications, including wound and burn treatment [1]. They exhibit effective traits that create significant potential in various fields, leading to extensive research efforts for their productization. In this context, many developments in the utilization of silver ions have been made. One of the main technologies that has extended the use and efficiency of silver ions is nanotechnology. Silver NPs are composed of multiple elemental silver atoms that are combined together to form an NP structure. These particles can abundantly release silver ions through oxidation, which enable their applicability in various areas. Silver NPs have been highlighted for their superior properties, such as antibacterial, antiviral, and anti-inflammatory activities [2,3]. Additionally, they exhibit unique physicochemical properties related to surface area, charge, shape, and localized surface plasmon resonance (LSPR), significantly affecting their biological activities [4]. Silver NPs can have varying sizes, from 1 to 100 nm, offering different attributes and uses. Size has been known to play a critical role in determining NPs’ efficiency, especially in sensing and imaging applications, considering its influence on LSPR, a phenomenon known to characterize silver NPs’ optical traits [5].

Similar to size, silver NPs can be found in various shapes depending on the parameters of synthesis methods (temperature, pH, concentration, etc.), yielding nanorods, nanoprisms, nanospheres, nanocubes, nanowires, and so on [6]. Each of these shapes can exhibit slight differences in their properties that alter their applications. For example, triangular-shaped silver NPs are highly valued in antibacterial applications for their sharp edges and vertices, which facilitates an improved interaction with bacterial cell membranes [7].

**Figure 1:**
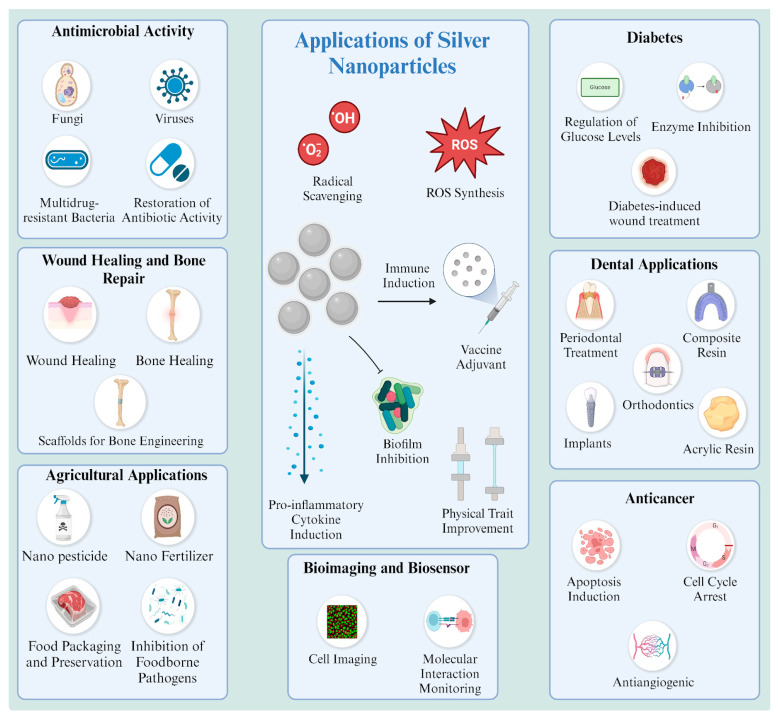
Applications of silver NPs that are covered in this review [10,11].

This is why the applications of silver NPs extend across a wide range of fields based on their unique characteristics. Along with other metal NPs, they can be applied in various fields, including but not limited to biosensor devices, anticancer applications, food packaging and preservation, antimicrobial activity, and wound healing (Figure 1) [8,9].

Several key areas that cover significant aspects of silver NP applications in the biomedical field are evaluated in this review. It needs to be emphasized that the antimicrobial activity of silver NPs is the central characteristic in biomedical applications. The sub-sections in the agricultural section discuss applications that predominantly utilize antimicrobial activity by inhibiting microbial growth for food preservation and preventing the growth of pesticides in plants. Additionally, some processes positively influence the physical attributes of synthesized food packages and the growth factors of tested plants. A similar correlation exists for dental and wound healing applications. Both of these areas require materials that exhibit significant antibacterial and antibiofilm activity. This is why current studies still use silver NPs in specific concentrations to control microbial growth in oral and wound areas, yielding additional positive results. Furthermore, NPs possess unique characteristics in certain specialties, such as cancer and diabetes research. The structural functionalization of silver NPs is also an important feature, especially in bioimaging and biosensor applications.

**Figure 2:**
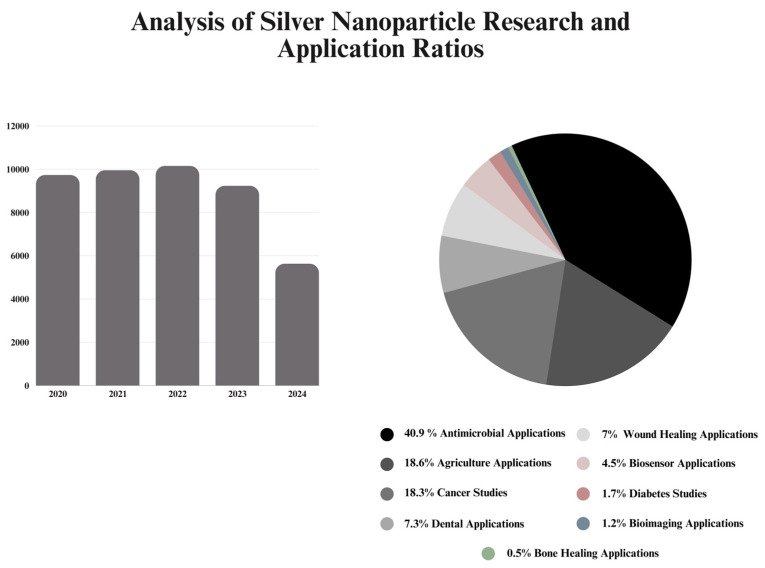
Graph representing the published research papers that include “Silver Nanoparticles” in their title between 2020 and 2024, with a pie chart representing the distribution of these studies based on the discussed applications [12].

The current literature represents a high intensity of research articles on “silver nanoparticles” (Figure 2). For the last few years, published research articles have shown steady numbers, demonstrating a graph plateau. By the end of 2024, there is a great possibility that the graph “plateau” will be preserved. Considering the distribution of these papers, we created a pie chart based on the discussed areas of applications, considering the total number of papers on “silver nanoparticle applications”. As expected, and highlighted in this review, antimicrobial-based application papers are predominant in the recent literature. Agricultural and cancer-based applications show a high ratio compared to other areas. Wound healing and dental applications share a similar ratio; the rest of the areas come afterwards, showing a small percentage in comparison.

Silver NPs have demonstrated promise in antimicrobial research against drug-resistant bacteria and oncology as anticancer agents. They elicit specific cytotoxicity, creating novel opportunities in cancer treatment. Research on wound healing indicates that silver NPs stimulate tissue regeneration and inhibit infections, showcasing notable progress in medical therapies. Moreover, they are employed in bioimaging and biosensing, especially in diagnostics, owing to their pronounced SPR and magnetic characteristics, rendering them essential for early illness identification. In this comprehensive review, the diagnostic and therapeutic uses of silver NPs, emphasizing less studied domains such as bone regeneration and diabetes research, are discussed. This research uniquely addresses the toxicological risks and regulatory challenges related to silver NPs, providing a forward-looking view of their safe integration into clinical practice. By categorizing and assessing current papers and patents across several fields (antimicrobials, agriculture, cancer, wound healing, dentistry, etc.), this research synthesizes trends and contextualizes silver nanoparticle use across sectors. Together with an analysis of the current literature on this research area, the results forecast ongoing growth and innovation for silver NPs, propelled by interdisciplinary cooperation and sustainable development. These NPs are anticipated to propel progress in nanotechnology and innovative medicinal therapies. However, their potential must also be assessed considering their environmental and ethical implications.

Despite the long-term study background of silver NPs, their research still has not shown any sign of a significant reduction in published study numbers. Considering the rise of antimicrobial applications in wide-ranging areas, it can be concluded that there will be many important applications of silver NPs. Since nanotechnology is still under development and many innovative approaches are still desired, silver NPs will remain highlighted, with great possibilities to be explored. This is why reviews on silver NP applications are extremely important to show guidance for the future of this area. In summary, we briefly cover most of the important areas, which are discussed in the following sections and shown in Table 1 to represent the recent findings in these areas.

## 2. Antibacterial Activity and Antibiotic Resistance

**Figure 3:**
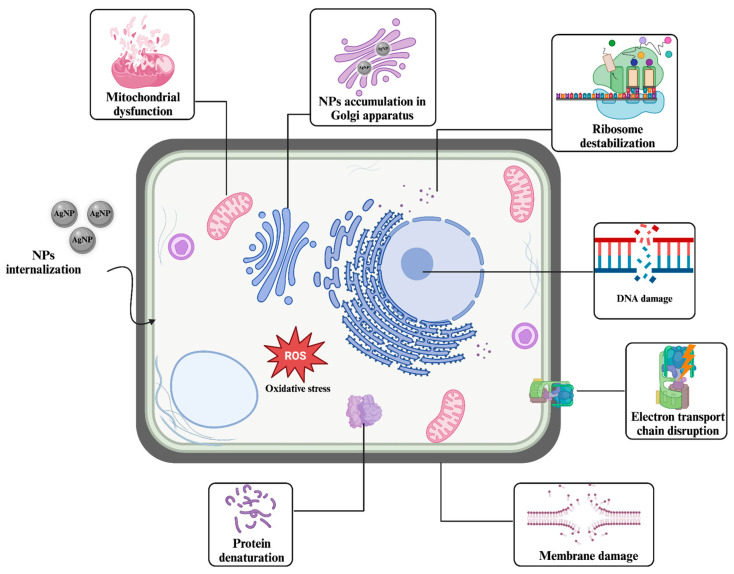
Antibacterial mechanisms of silver NPs [86].

Silver has been used for many purposes, particularly in antibacterial applications, for a long time [85]. With the advancement in and preference for NP technology, silver has become one of the primary materials in nanotechnology. The most notable characteristic of silver NPs is their multi-directional mechanisms against bacteria. Some of their diverse potential mechanisms damage DNA and proteins by increasing ROS levels, disrupting cellular membranes by accumulation, and interfering with antibiotic resistance mechanisms [86] (Figure 3).

The structure of silver NPs is a key physicochemical property that influences their interaction with bacterial cells. Small-sized, plate-like silver NPs can exhibit the strongest surface binding through their large surface area to ensure the efficient release of silver ions [87]. To obtain the high silver ion release, surface stabilization should also be considered to avoid aggregation among silver NPs, which reduces the surface area [5,88]. 

Variations in the antibacterial activity of silver NPs depending on their shape can be observed in various studies. For example, a study tested three different shapes of silver NPs (strawberry-like, yolk–shell, and cable-like nanofibers) modified with polyphosphazene against *Escherichia coli* (*E. coli*) and *S. aureus* [89]. Although all three types of NPs demonstrated significant bactericidal activity, the strawberry-like silver NPs exhibited the strongest activity, showing the lowest MIC and minimum bactericidal concentration (MBC) compared to the other two structures. The strawberry-like silver NPs demonstrated MIC values of 39.4 µg/mL for *E. coli* and 312.5 µg/mL for *S.aureus*, while the values were 153.6 and 625 µg/mL for cable-like nanofibers and 625 and 1250 µg/mL for yolk–shell NPs, respectively. The difference in MBC values was more severe as the yolk–shell silver NPs showed the highest concentrations. The authors noted that strawberry-like silver NPs exhibited a balanced distribution of silver ions through the polyphosphazene shell layer, ensuring direct contact with bacterial cells and efficient ion release. 

A similar study tested four types of silver NPs (spherical, rod, triangular, and hexagonal) against various Gram-positive and Gram-negative bacteria [90]. All types of silver NPs were tested at various concentrations and their zones of inhibition and MIC values were observed. At concentrations of 242 and 249 µg/mL, silver nanospheres showed the most significant zone of inhibition while requiring the lowest concentration compared to other structures. In terms of MIC values, silver nanospheres showed the lowest concentration, with only a slight difference compared to the other three types. The amount of released silver ions was also determined: nanosphere silver NPs demonstrated the highest silver ion release at 34 µg/mL, silver nanorods released 32 µg/mL, nanotriangle-shaped silver NPs released 26 µg/mL, and hexagonal silver NPs showed the lowest release at 15 µg/mL.

Many similar studies compare the antibacterial activity of different silver NP structures. One study compared three types of silver NPs (nanocubes, nanospheres, and nanowires) and demonstrated that nanospheres and nanocubes had significantly greater antibacterial activity compared to nanowires, likely due to their larger specific surface areas [91]. Another study demonstrated that dendritic-shaped silver NPs had stronger antibacterial activity than spherical ones [92]. The influence of the physicochemical properties of NPs on their applications is well known. This holds true for silver NPs, especially in terms of their antibacterial activities. NPs with higher surface areas and efficient silver ion release will yield better results in antibacterial applications.

Numerous studies have been conducted on the antibacterial activity of silver NPs. This is because, along with their bactericidal and bacteriostatic activity, silver NPs also exhibit significant efficiency against biofilms [93]. Current antibacterial applications of silver NPs are highly oriented towards green synthesis methods. For example, numerous leaf extracts [94], along with various types of algae, bacteria, and fungi [95], have been used for silver NP synthesis and tested on different bacteria for antibacterial activity. Including all studies of this variety would lead to repetitive statements in discussing silver NPs’ antibacterial activities. This is why we focused on antibacterial activity against multi-drug-resistant bacteria and synergistic activity with antibiotics. In this way, the antibacterial activity of silver NPs is covered since most of the studies discussed below solely demonstrated antibacterial activity. NPs’ influence on antibiotics is also highlighted. Additionally, we included certain recent studies in Table 1 to emphasize the antibacterial activity.

Silver NPs are widely applied to multi-drug-resistant bacteria, both with and without antibiotics. It has been proposed that silver NPs are highly effective against drug-resistant bacteria and significantly lower resistance buildup. Excessive use of antibacterial drugs and antibiotics, especially with poor administration and efficiency levels, increases the chance of forming drug-resistant biofilms [96]. These biofilms can be composed of multiple strains and spread among other organisms in the biofilm once resistance develops. Therefore, silver NPs are extensively used as an alternative for dealing with antibiotic-resistant bacteria by reducing the administered drug amount and utilizing NP-based antibacterial activity [97]. For instance, green synthesized silver NPs were tested on three types of bacteria, including antibiotic and multi-drug-resistant strains: *Enterococcus faecalis* (*E. faecalis*), *Pseudomonas aeruginosa* (*P. aeruginosa*)*,* and *Acinetobacter baumannii* (*A. baumannii*) [98]. The antimicrobial activity of silver NPs was tested using a well diffusion assay to determine the inhibition zone for each bacterium. In each group, the antibiotic or drug to which the bacteria were resistant was used as a positive control group. As expected, the non-resistant strains showed significant inhibition zones of 27 mm, 25 mm, and 16 mm, respectively, while the resistant strains showed similar results to the negative control (6 mm). Regarding the activity of silver NPs, a large inhibition zone was observed for all bacteria, including resistant strains. The inhibition zone for non-resistant *E. faecalis* was almost the same as for its resistant strain, 12.0 to 12.3. Meanwhile, the inhibition zone for non-resistant *P. aeruginosa* was slightly larger than for its resistant strain, 19.8 to 16.3. Conversely, the inhibition zone for resistant *A. baumannii* was slightly larger than for its non-resistant strain, 17.7 to 16.8. These results were obtained at the highest silver NP concentration (360 μg/mL), but these correlations held true at almost all other concentrations, with an observable increase in inhibition zones becoming clear at concentrations of 45 μg/mL and higher. MIC and MBC assays were also performed. Except for resistant *A. baumannii* (11.25 μg/mL), the MBC value was the same for all bacteria at 22.5 μg/mL. Similarly, MIC values were only different for both resistant and non-resistant *E. faecalis* (11.25 μg/mL), with the rest showing 5.6 μg/mL. These results indicate that silver NPs can exhibit antibacterial activity independently of antibacterial/drug resistance.

Although silver NPs can exhibit bactericidal activity against antibiotic-resistant bacteria, they can also be synergistically administered with distinct antibiotics against multi-drug-resistant bacteria. They can also make bacteria susceptible to antibiotics or drugs to which they were previously resistant. For instance, a comprehensive study demonstrated the enhanced antibacterial activity of silver NPs combined with 14 different types of antibiotics against seven distinct pathogens [99]. Multiple enhancement results were observed with the combination of silver NPs, such as a 3.0-fold increase in the activity of penicillin against *Streptococcus mutans* (*S. mutans*), 1.8-fold increase in the activity of amoxicillin against *P. aeruginosa*, and 3.8-fold increase in the activity of vancomycin against *Enterobacter aerogenes*. Most importantly, several multi-drug-resistant bacteria became susceptible to multiple antibiotics, such as vancomycin-resistant *S. mutans* and multidrug-resistant *A. baumannii*. Understanding the mechanisms that bacteria develop for resistance to specific antibiotics is extremely important for understanding the synergistic relationship between silver NPs and antibiotics.

A study investigated the combined activity of silver NPs with certain antibiotics against antibiotic-resistant *A. baumannii* [100]. Both solo and combined treatments of silver NPs significantly suppressed the growth of the bacteria, both *in vivo* and *in vitro*. Additionally, they proposed a potential mechanism behind this synergistic activity between the NP and antibiotics by using *E. coli* as a model system; silver NPs could enhance polymyxin B-induced membrane lipid damage and bind intracellular proteins and RNA polymerase with Rifampicin. A similar study investigated the enhancement of aminoglycosides by silver NPs against antibiotic-resistant bacteria [101]. It was demonstrated that the addition of silver NPs decreased the MIC of aminoglycosides by up to 22-fold, including amikacin. Other noteworthy examples include tobramycin (19-fold), gentamicin (12-fold), and colistin (6.9-fold). Except for colistin, which targets the outer membrane, all other antibiotics that showed significant fold changes were ones that target ribosomal subunits. This suggests that these aminoglycosides, along with silver NPs, can target ribosomal subunits to inhibit bacteria. Additionally, the fold change of tetracycline, which also targets the ribosomal subunit, was only 2.2, suggesting additional factors behind this synergistic activity.

In addition to their synergistic activity, silver NPs can remove the resistance factor from bacteria and restore an antibiotic’s activity through several potential mechanisms. For instance, a study investigated the effect of silver NPs on the efficiency of four different antibiotics against three types of *Burkholderia pseudomallei* strains. Similar to the discussed studies, both silver NPs alone and in combination with different types of antibiotics showed significant antibacterial activity. However, one highlighted finding was the resistance of isolate 316c to ceftazidime. When this antibiotic was administered without silver NPs, the MIC and MBC values were significantly higher, at 128 µg/mL and 512 µg/mL, respectively. When the silver NPs were added, the values were extraordinarily lowered to 16 µg/mL for both, indicating an 8-fold and 32-fold decrease in concentration, respectively. At the same time, all three strains (H777, 1026b, 316c) showed resistance to gentamicin, with MIC values of 16, 32, and 64 µg/mL and MBC values of 256, 512, and 512 µg/mL, respectively. These values were significantly lowered when these antibiotics were administered along with silver NPs by 4- to 16-fold for MIC values and 32- to 512-fold for MBC values. These extreme changes in MIC and MBC values indicated that silver NPs restored the activity of these ineffective antibiotics against resistant strains. The mechanism behind this activation effect is not clear beyond the fact that silver NPs can enhance the activity of antibiotics. However, there may be additional mechanisms behind these extreme value changes in concentrations. Bacteria can develop resistance to an antibiotic through several mechanisms, including the removal of antibiotics by efflux pumps, modification or degradation of the antibiotic by enzyme catalysis, and the synthesis of decoys to divert the interaction of antibiotics from their original target [102]. It has been proposed that silver NPs can increase antibiotic uptake by disrupting cell walls through ROS synthesis, protecting antibiotics against bacterial enzymes (if attached to the NPs), and the blocking/downregulating of efflux pumps by silver NPs [103]. For example, it has been demonstrated that silver NPs can exhibit efflux pump inhibitory activity against multidrug-resistant *A. baumannii* [104]. Along with the antibacterial activity of silver NPs, the potential inhibitory effect on efflux pumps was tested using the ethidium bromide (EtBr) cartwheel method. Each isolate’s MIC values were calculated with sole EtBr and silver NP treatment and compared with the combined treatment of the two. Each isolate showed at least a 2- to 4-fold change in MIC value, indicating significant potential for inhibitory activity. In addition, significant downregulated efflux-related mRNA levels were also observed, potentially due to the silver NP-generated ROS. Another study showed that the multi-target ability of silver can restore the sensitivity of *S. aureus* to methicillin antibiotics [105]. First, the main mechanism behind the antibacterial activity of silver ions (compared with silver NPs) was identified, which involves the rapid targeting of proteins involved in glycolysis, the ROS defense system, and the oxidative pentose phosphate pathway. Additionally, it was revealed that the addition of silver ions significantly reduced the concentration of the extremely resistant strains, whereas antibiotic treatment alone did not. Finally, considering the significant decrease in the MIC value of ampicillin, it was confirmed that silver ions managed to restore the sensitivity of bacteria to the antibiotic. Since silver targets multiple pathways simultaneously, similar to silver NPs, it is likely that this unique feature is the main mechanism behind the removal of antibiotic resistance.

These results might indicate an additional mechanism behind the synergistic activity between silver NPs and antibiotics. The synergistic activity is promising and holds great potential for future antibacterial research, but disrupting antibiotic resistance and restoring the efficacy of antibiotics is more critical. Investigating these potential interactions will certainly contribute to addressing the current worldwide problem of antibiotic resistance. In addition, discovering new insights into silver NPs will surely enhance their application in these areas.

Along with general antibacterial mechanisms, it is clear that silver NPs cover a large proportion of antibacterial research. Their antibacterial activity is unquestionable and supported by extensive experiments, but it is not the area that should be focused on to develop their impact on these research areas. If silver NPs are considered one of the primary candidates for dealing with antibiotics and drug-resistant bacteria, their interaction with bacterial mechanisms and other antibiotics should be examined.

## 3. Antiviral Applications

**Figure 4:**
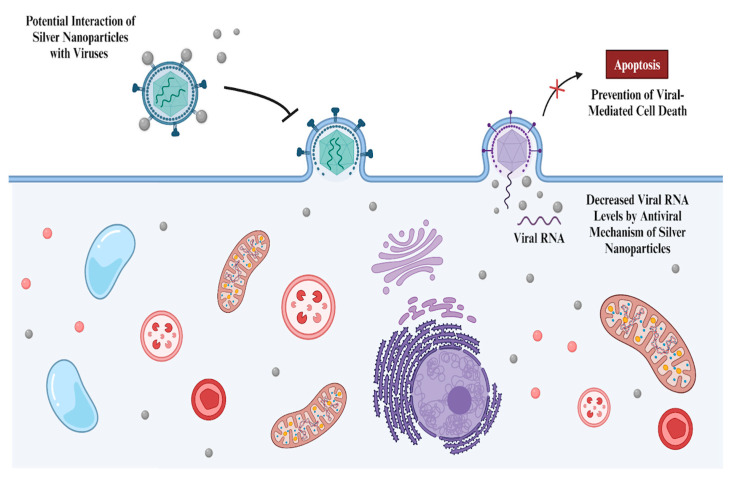
Antiviral mechanisms of silver NPs [108].

Similar to their antibacterial characteristics, silver NPs also possess antiviral mechanisms. These mechanisms involve potential intracellular and extracellular actions (Figure 4) [106], depending on the type of virus and the properties of the NPs, which will be discussed in the next paragraph. Size emerges as an important property that is capable of influencing NPs’ virucidal effect. Aschalew *et al.* showed that silver NPs exhibit size-dependent antiviral activity against feline calicivirus (FCV), with 10 nm NPs demonstrating the greatest inhibition in comparison to their larger counterparts, 75 nm and 100 nm. They stated that 10 nm silver NPs effectively inhibited FCV, beyond the limit of detection, and led to a 6.5 log_10_ reduction in viral titer, while larger NPs did not show comparable results [107]. Silver NPs are also coated with different materials for antiviral applications against specific viruses, such as hepatitis B, human immunodeficiency virus type 1, and herpes simplex virus type 1 [108]. For instance, an *in vivo* study was conducted to demonstrate the antiviral activity of silver NPs against the respiratory syncytial virus [109]. An *in vitro* experiment was conducted on epithelial cells to show the dose-dependent activity (a 50 µg/mL concentration exhibited the highest activity) of silver NPs, resulting in a significant 79% decrease in viral replication. Subsequently, silver NPs demonstrated antiviral activity in an *in vivo* experiment, showing 55% and 45% decreases in viral copy numbers at 4 mg/kg and 2 mg/kg, respectively. Additionally, silver NPs also reduced cytokine and chemokine levels, which were increased by infection. The levels of neutrophils were maintained and recruited by the silver NPs. Another study tested the antiviral activity of silver NPs against SARS-CoV-2 [110]. Both polyvinylpyrrolidone-capped silver NPs and colloidal silver were tested against SARS-CoV-2. The *in vitro* study demonstrated that both the silver NPs and colloidal silver significantly decreased viral RNA copy levels and prevented viral-mediated cell death, depending on size and concentration, respectively. Silver NPs between the sizes of 2 and 15 nm showed the most efficient antiviral activity. Besides the intracellular effects, silver NPs also exhibited extracellular activity. The antiviral activity was observed by introducing silver NPs into the culture at different infection phases. Adding both the virus and the NP showed a significant reduction in viral RNA copies and the highest rate of survival of the cell, indicating the extracellular activity along with intracellular inhibition. Treatment with silver NPs after the infection phase showed both types of inhibition mentioned above, but the efficiency was significantly lower. Early treatment with silver NPs before infection showed the lowest efficiency. Even though not completely understood, silver NPs may have size-dependent molecular interactions with SARS-CoV-2 (targeting the virus itself or a specific receptor that is involved in cellular attachment, like ACE-2) and the ability to bind intracellular molecules, similar to their antibacterial activity, which reduces RNA viral copies.

Silver NPs possess similar mechanisms behind their antiviral activity. There is no doubt that these mechanisms are highly influenced by the size and surface properties of the NPs, which are major factors in antimicrobial application. Similar antiviral mechanisms, related to changes in cytokine levels and the inhibition of viral infectivity, are also observed in other types of viruses such as human parainfluenza virus type 3 [111], herpes simplex virus type 2 [112], and human immunodeficiency virus type 1. The antimicrobial activity of silver NPs, generally exhibiting similar mechanisms, is applied to a distinct type of bacteria (in addition to those discussed in the previous section), and certain species of fungi as well [113]. Simultaneously, the effect of physicochemical properties is also discussed for other antimicrobial applications of silver NPs. While the mechanisms behind the antimicrobial activity of silver NPs against various microorganisms are similar, the impact of physical properties becomes just as important as in antibacterial and antiviral activity. Thus, certain small-sized shapes of silver NPs can be more effective against fungi than larger-sized particles [114].

Silver NPs carry significant potential in antimicrobial applications, and a substantial number of studies are currently present in the literature. Studies focus on comparing various parameters, such as the source, size, surface properties, concentrations, and different modifications of silver NPs, rather than directly observing activity efficiency. These characteristics of silver NPs certainly comprise one of the major areas in NP applications. Additionally, the antimicrobial activity of silver NPs is not solely limited to direct investigation. As will be discussed in the following sections, these mechanisms are highly utilized in other areas as well.

## 4. Agricultural Applications

Foodborne pathogens pose a significant threat to public health as they are responsible for many foodborne diseases [115]. Additionally, they pose a great risk to the quality and taste of food, which damages the food market. To overcome this growing problem, many technologies have been integrated into these areas. Currently, one of the most studied technologies in food applications is nanotechnology. Packaging needs to be suitable for preserving food and its quality, as well as preventing any external contamination and controlling microbial growth [116]. In these cases, many nanotechniques, especially the use of NPs and nanocomposites, are widely used in food packaging and preservation applications [117].

**Figure 5:**
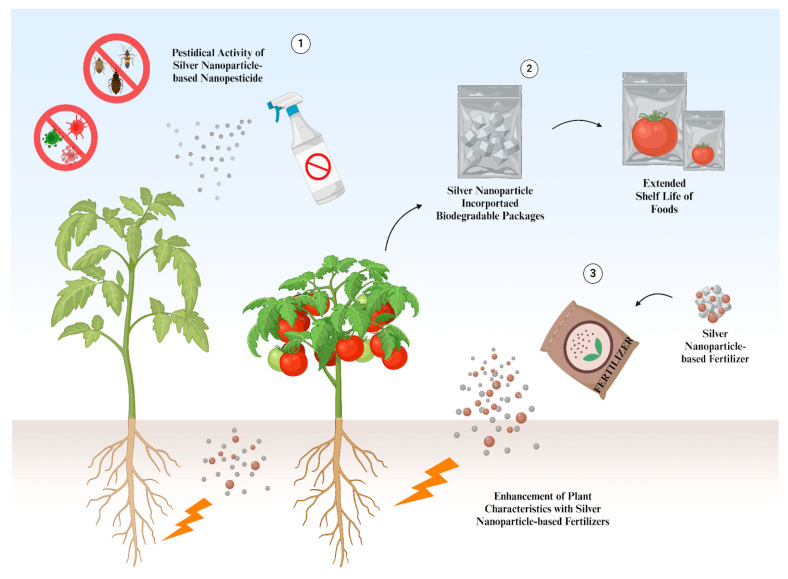
Agricultural applications of silver NPs. (1) Silver NPs can be used as nanopesticides for pesticidal activity. (2) Silver NPs can be incorporated into food packages to preserve and increase the shelf life of foods. (3) Silver NP-based fertilizers can enhance the characteristics of plants [125].

In packaging and food preservation, antimicrobial characteristics are very important for decreasing food contamination and improving shelf life. This is why antimicrobial packaging is effective and has the potential to deal with microbial contamination of food products [118]. Due to their antimicrobial activity, silver NPs have been included in many food packaging and preservation studies for diverse food types (meat, peppers, strawberries, cheese) against diverse microorganisms (foodborne pathogens, yeasts, molds) [119]. Many silvers NP-based materials have been used for packaging to improve physical and antibacterial properties (Figure 5) [120]. In most of these applications, silver NPs are either hybridized with other types of NPs or coated with different materials to achieve maximum efficiency in the application.

Chitosan is a polymer that possesses powerful antimicrobial activity and is included in many composites for food packaging applications [121]. In addition to its antimicrobial activity, it exhibits great antioxidant capacity, enzyme inhibition, and biodegradability [122]. These attributes of chitosan have been studied for a long time and highlighted for food packaging applications. Currently, certain nanomaterials are combined with chitosan to enhance packaging applications. Silver NPs are among the most common nanomaterials utilized with chitosan for dealing with foodborne pathogens. For instance, a composite film based on silver NPs, chitosan, and gelatin was developed for biodegradable food packaging applications [123]. First, the antioxidant activity (up to 69% scavenging activity at higher concentrations) and antibacterial activity of silver NPs were demonstrated against five types of bacteria: *P. aeruginosa*, *E. coli*, *S. aureus*, *S. mutans*, and *Candida albicans* (*C. albicans*). In addition to providing antibacterial activity, silver NPs enhanced the physical properties of the composite film by increasing tensile strength (TS), decreasing swelling degree through the cross-linking action of the NPs, and reducing the water vapor transmission rate and moisture retention capability. To test the potential application of this composite film with antibacterial activity, carrot pieces were used. By storage day 10, the carrot pieces had turned a black color due to microbial activity, whereas the composite film samples maintained their color and showed no structural changes, indicating no foulness or decay. Lastly, the colony forming unit value of the carrot pieces was observed using the spread plate method and was 8-fold lower for the composite film samples. A recent study also prepared a silver NP and chitosan antibacterial film with polyvinyl alcohol for food packaging applications [124]. Similarly, the addition of silver NPs (at a moderate concentration) increased the TS and water resistance of the film and decreased water WVP, while chitosan and polyvinyl managed to increase the stabilization of the NPs. Antibacterial activity was also demonstrated on *E. coli* and *S. aureus*, showing superior results. Finally, the antibacterial film was tested on strawberries. It was found that the weight loss of the strawberries during storage was significantly lower with the silver NP film, preserving their color and preventing decay up to day 9.

In addition to food packaging, there are various sub-areas of agriculture where silver NPs are widely utilized. They are one of the efficient inorganic NPs that are used as nano fertilizers since they exhibit significant antimicrobial characteristics for plant diseases [126]. They have been used on different types of crops to increase heat stress tolerance, enhance growth and root nodulation, and increase microbial diversity while inhibiting pathogenic bacteria [127]. Along with their efficient management of plant diseases, applications of silver NPs, especially their biosynthesized forms, as biofertilizers can reduce the usage of chemical fertilizers in unnecessary amounts, presenting a more environmentally friendly approach. As an example, researchers carried out a field experiment with silver NPs containing nitrogen fertilizer [128]. The germination of crop seeds (barley, peas, rapeseed) and vegetable seeds (radish, cucumber, lettuce) was investigated in the presence of silver NPs and a urea solution. For crop seeds under habitat conditions, except for the increased length of the rapeseed sprout, there was no significant change in any germination feature in the presence of silver NPs. However, the NPs managed to decrease infection in pea seeds and increased germination energy and the length of barley and rapeseed sprouts under thermal stress. Pathogen contamination was also reduced under stress conditions for all seeds as well. Certain germination characteristics of vegetable seeds under thermal stress were improved in the presence of silver NPs. Sprout length and germination energy were increased; meanwhile, the number of abnormal sprouts was decreased for cucumber. The seed germination and energy of radish were also positively influenced by NP treatment. Pathogen infestation was significantly reduced for all vegetable seeds, even under optimal conditions for cucumber and lettuce. Finally, the chlorophyll content also increased.

Silver NPs also positively influence plant growth in other conditions. For instance, the effect of silver NPs on plants under salt stress was investigated in pearl millet [129]. Silver NPs significantly improved the morphological and physiological attributes of the plant with and without salt treatment: 150 mM NaCl showed the most inhibitory effect on the plant and 20 mM silver NPs showed the most efficient result by increasing plant length, shoot dry weight, and grain weight (between 21% and 23%). Chlorophyll concentrations slightly increased and hydrogen peroxide levels were significantly reduced. A similar study also showed the activity of green synthesized silver NPs on spinach (*Spinacia oleracea* L.) under salt stress [130]. Seed germination and plant growth were observed with the treatment of silver NPs at various concentrations (0, 20, 40, 80, and 100 ppm) under four salinity levels (0, 50, 100, and 150 mM NaCl). The researchers showed that silver NPs increased the salt tolerance of seedlings under high saline concentrations. In addition, certain parameters of spinach, such as growth parameters, vigor index, and chlorophyll contents, were increased by silver NP treatment. These results highlight the usability of silver NPs in agricultural applications where plants suffer from saline environments.

Additionally, silver NPs can potentially influence various biochemical parameters related to plants, i.e., micro- and macronutrients. In a recent study, Tokarz *et al.* developed an approach using silver NPs in a foliar application to increase the nutritional value of potato tubers [131]. The administration of NPs led to improvements in the properties of potatoes in a dose-dependent manner, with lower concentrations leading to enhanced antioxidant activity by increasing the content of phenolic compounds and free radical scavenging efficiency, while higher concentrations resulted in increased levels of macro- and micronutrients, ascorbic acid, and soluble sugars. Hence, careful consideration of NP dosage is required to fully optimize and improve their utilization in agricultural applications.

Lastly, some studies investigated the application of silver NPs in agriculture for controlling weeds and pests. For instance, the herbicidal activity of silver NPs against *Bidens pilosa* (*B. pilosa*) L. was investigated [132]. The germination of *B. pilosa* was studied for 7 days after cultivation. Treatment with silver NPs inhibited and delayed the growth of *B. pilosa* by decreasing the germination rate from 52.22% (water treatment group) to 14.44% in 3 days (the germination rate was 18.64% after 7 days in the silver NP treatment). Root and shoot elongation of the *B. pilosa* was also investigated, with silver NPs inhibiting it by 19.38% and 23.33%, respectively. Similarly, the nano-pesticidal potential of silver NPs was demonstrated on phyto-pathogens of tomato [40]. Several antimicrobial features of silver NPs were observed during the experiments. The antibacterial activity was tested against the plant bacterial pathogen *Pseudomonas syringae*. The NP-treated bacteria showed great morphological changes on the surface and increased membrane permeability due to cellular membrane damage. Biofilm formation was also prevented. Later, antifungal activity was demonstrated on three types of fungi that are majorly responsible for damaging crops: *Rhizoctonia solani*, *Sarocladium* sp., and *F. oxysporum*. Silver NPs exhibited a dose-dependent inhibition of mycelial growth against all three pathogens. Additionally, the inhibitory effect of silver NPs on *Meloidogyne incognita* was demonstrated by inhibiting hatching and increasing nematode mortality. Finally, these features were tested on tomato crops by separately infecting them with bacteria, fungi, and nematodes. It was observed that silver NPs enhanced the seedling development, biomass, and growth parameters of tomatoes when infected. A high antioxidant response was also observed against each type of pathogen.

Silver NPs hold great potential in agricultural applications. Most of their applications are based on their antimicrobial characteristics, along with their structural features as NPs. They also show additional improvements in these applications, such as providing physical support in food packaging and enhancing certain features of plants. Considering the diverse applications of NPs in agriculture, it can be beneficial and eco-friendly to investigate and develop the application of NPs (including silver NPs) in agriculture, which can positively influence human health in the future.

## 5. Wound Healing Applications

**Figure 6:**
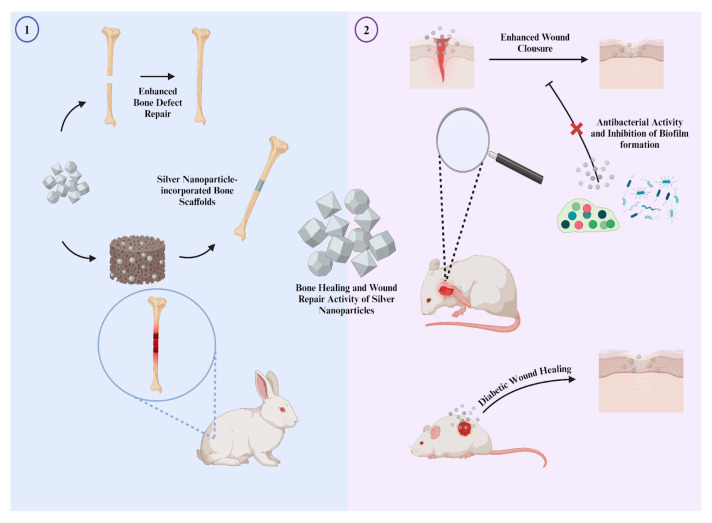
Bone healing and wound repair activity of silver NPs. (1) Silver NPs enhance bone defect repair progress and can be included in bone scaffolds. (2) Silver NPs enhance the repair of chronic and diabetic wounds. They also possess antibacterial and antibiofilm activities during wound healing [47,135].

Wound healing applications are another area that utilizes the antimicrobial activity of silver NPs. Generally, inorganic NPs that exhibit antimicrobial activity are combined with polymers to prevent microorganisms from growing at wound sites [133]. In addition to polymers, many other molecules are combined with silver NPs in various forms, such as hybrid NPs and nanocomposites. These combinations are widely used because they have been tested on certain types of wounds, such as diabetic and chronic wounds, and commercialized for wound treatment [134]. Thanks to their significant antimicrobial activity and the extensive research in the current literature, silver NPs are increasingly used in wound healing applications (Figure 6).

Rigo *et al.* demonstrated the wound healing properties of active silver NP-based dressings on 3D fibroblast cell cultures [136]. The effect of silver NPs on mitochondrial activity was observed and found to be negatively influenced, with a significant decrease after day 3. Conversely, they found no trace of damage to nuclei or nuclear fragmentation and observed an increase in the number of live cells in dermal-like tissue. They combined these findings with other references to hypothesize that the impact of silver NPs on mitochondrial activity was independent of cytotoxicity and cell death. Furthermore, the distribution and release of silver ions were studied. They found that most of the silver ions (approximately 94%) remained in the dressing. The research was extended to an *in vivo* experiment on a single patient. The dressing successfully restored the structure of the biological tissue, and the wound healed after 10 additional days of treatment. The regrowth of the tissue structure was confirmed through optical microscopy, possibly indicating epidermal repair. The patient was fully healed by day 17, without any adverse effects of the silver NPs inside the tissue and cells. Transmission electron microscopy images showed that silver NPs were mainly localized in the cytoplasm, with no traces inside the nucleus. Additionally, the authors speculated that the number of mitochondria increased and repositioned themselves to protect the nucleus from possible threats posed by the silver NPs, acting as a physical and chemical barrier. These findings indicate that long-term administration of silver NP dressing did not pose any significant threat and did not interfere with the proliferation of fibroblasts and wound healing processes.

Similarly, a study was conducted to test the antimicrobial and wound healing activity of green synthesized silver NPs on cotton fabrics in fibroblast cells [137]. The antimicrobial activity test showed that silver NPs significantly inhibited the tested pathogens: *P. aeruginosa, S. aureus*, *S. pyogenes*, *C. albicans*, and *E. coli*. The antimicrobial activity experiment was repeated on the same pathogens, but this time with silver NPs loaded onto cotton fabrics. None of these pathogens managed to grow on the cotton fabrics. An *in vitro* experiment was then conducted on fibroblast cells to examine the wound healing potential. The cell migration ratio was significantly increased at 15 μg and 20 μg. The proliferation of fibroblast cells was significantly enhanced at certain concentrations without any observable cytotoxicity, except when the concentration reached 35 μg.

Since silver NPs do not pose a threat to wound healing applications and exhibit the desired characteristics for regulating wound healing, many silver NP-containing hydrogels have been developed to enhance wound healing activity. Research demonstrated the antibacterial activity and wound healing activity of silver NP-loaded PF127 polymer hydrogel in mice [138]. Silver NPs alone exhibited radical scavenging activity between 33.73% and 65.17%, depending on the concentration. The antibacterial activity was tested with both silver NPs and hydrogel and was significant for both samples. An *in vivo* study was conducted to examine the wound healing activity of the hydrogel and showed promising results without any skin irritation. The control group had a wound healing rate starting from 23.12% on day 3 to 60.12% on day 10 (the day the wounds were closed). These rates were significantly enhanced in the hydrogel-treated group with two different concentrations, 0.3 mg and 1.0 mg. Until day 7, the concentration did not significantly alter the wound healing ratio. However, the healing ratio was 85.52% for the 0.3 mg hydrogel group and 94.54% for the 1.0 mg hydrogel group. A similar study synthesized guar gum/curcumin-stabilized silver NP hydrogel composites for wound dressing materials [139]. The curcumin–silver NP composite did not induce any cytotoxicity at certain concentrations (below 0.100 nM) and exhibited viable cell viability at 0.200 nM with an 80% cell rate. In addition, it managed to enhance proliferation and collagen production in the fibroblasts by up to 45% and 50%, respectively. *In vitro* experiments also revealed that the curcumin-silver NP composite at low concentrations greatly reduced wound gaps and enhanced cell migration over time. Moreover, the *in vivo* experiment clearly showed the enhancement of wound healing and the antibacterial activity of the synthesized hydrogel in rats. The results were compared with commercial antibacterial wound gels, and hydrogels achieved 73% wound healing, compared to commercial gel activity with 51%. When considering the time factor, the hydrogel-induced wound repair was 40% faster than in the control group. The antibacterial activity comparison was similar, as silver NP hydrogel exhibited 60% higher activity than the commercial gel. Histopathology and gene expression levels were also considered to promote the effect of the hydrogel in wound healing applications. The histopathology results indicated that the hydrogel-treated group had a shorter inflammatory phase than the control group. The number of the fibroblast cells and percentage of re-epithelialization were higher as well. On the other hand, the mRNA levels revealed that interleukin-6 levels were significantly lower in hydrogel treatment, possibly related to histopathology results, indicating a shorter inflammatory phase. Collagen and epidermal growth factor expressions were significantly increased.

The use of silver NPs in wound healing applications clearly shows that silver NPs are among the most suitable candidates to enhance antibacterial activity during wound treatment. Not limited by this, silver NPs can be utilized with different compounds to further enhance the healing process by positively influencing fibroblast cells, accelerating healing, and regulating certain environmental factors through radical scavenging and affecting the expression of inflammatory molecules. Even though most studies indicate that silver NPs do not possess significant toxicity, their mechanism of interaction with cellular components needs to be revealed. Silver NPs will likely cover a larger space in wound healing applications.

## 6. Bone Repair Applications

Thanks to their rich characteristics, many studies aim to involve silver NPs in bone healing and regeneration applications to enhance the healing process and utilize their significant antimicrobial activity (Figure 6). In research, the interaction between silver NPs and mesenchymal stem cells (MSCs) is a primary focus. Key strategies involving silver NPs in bone healing applications include inducing osteoblast differentiation, enhancing MSC proliferation, incorporating silver NPs into nanocomposites for bone regeneration, and inducing anti-inflammatory properties in MSCs [140]. In this section, we evaluate several of these strategies with major examples and present additional studies in Table 1.

Numerous studies investigate the effect of silver NPs on bone healing. For instance, one study investigated the effect of silver NPs on MSCs and the mechanism behind bone fracture healing [141]. An *in vitro* experiment demonstrated the activity of silver NPs on mouse MSCs. Silver NPs managed to increase cell proliferation by nearly 2.5-fold at a specific concentration of 4 μM. Other concentrations did not affect cell proliferation. This specific concentration was also non-toxic to cells, as confirmed by cell viability tests. Later, cells were cultured in a control medium and differentiation medium and the alkaline phosphatase (ALP) levels were observed with and without the presence of silver NPs. On day 10, there was no significant change in alkaline phosphatase levels in any of the groups. However, on day 14, a significant increase in protein levels was observed in the differentiation medium group. In addition, silver NP groups with differentiation mediums at 15 and 20 μM showed further increased protein levels. The experiment also revealed that silver NPs increased the expression of a regulatory gene that is responsible for osteoblast differentiation on day 14. It is noteworthy that the mRNA expression was equal between silver NP and control groups on day 18 and lower in the NP group on day 21. Overall, the results indicated the influence of silver NPs on osteoblast differentiation. Thanks to these positive results, the study carried into an *in vivo* experiment to observe the bone fracture healing activity of silver NPs in mice. The NPs were encapsulated in a collagen gel and administered at the bone fracture site. X-ray results showed that silver NPs nearly closed the gap by day 14 and fully closed it by day 21, while the fracture gap was still observable in the control group. Increased formation of new callus at the fracture site and promoted cell migration were demonstrated through histological analysis. Moreover, the authors found that silver NPs slightly induced TGF-β/BMP pathways, potentially related to the osteogenic differentiation of MSCs. Enhancement of bone healing by a silver NP composite was also demonstrated in a rabbit model, resulting in improved osteogenic properties [142].

The antibacterial activity of silver NPs is also utilized in bone healing applications. A collagen scaffold composite, incorporating silver NPs and bone morphogenetic protein 2 (BMP-2), was applied to infected bone, enhancing the repair process [143]. The antibacterial activity of silver NPs was demonstrated on *S. aureus*, while a scaffold without NPs showed no activity against the bacteria. Thereafter, the scaffold composite managed to slightly increase the proliferation of bone marrow-derived MSCs, either by silver NPs or BMP-2. More importantly, the scaffold significantly increased gene expression (RUNX2) and protein expression (ALP, osteopontin, and osteonectin). A similar study modified a silk fibroin/nanohydroxyapatite hydrogel with silver NPs and gold NPs to enhance antimicrobial activity for bone tissue engineering [144]. The experiments not only showed the significant antimicrobial activity of silver NPs but also highlighted their lower amounts of toxicity and compatibility with osteoblast cells.

Similar to wound healing applications, silver NPs are also compatible and do not pose any significant treatment issues in bone healing applications. Various models indicate that the possible intracellular activity of silver NPs positively affects bone healing pathways and cellular activities. The well-known antibacterial activity of silver NPs is also a significant advantage, especially for studying infected bone models. With further investigation of these potential mechanisms and involved pathways, silver NPs could be widely used in many scaffolds for bone healing and possibly bone engineering. The physical attributes of these scaffolds due to NP involvement should also be considered.

## 7. Vaccine Adjuvant Applications

**Figure 7:**
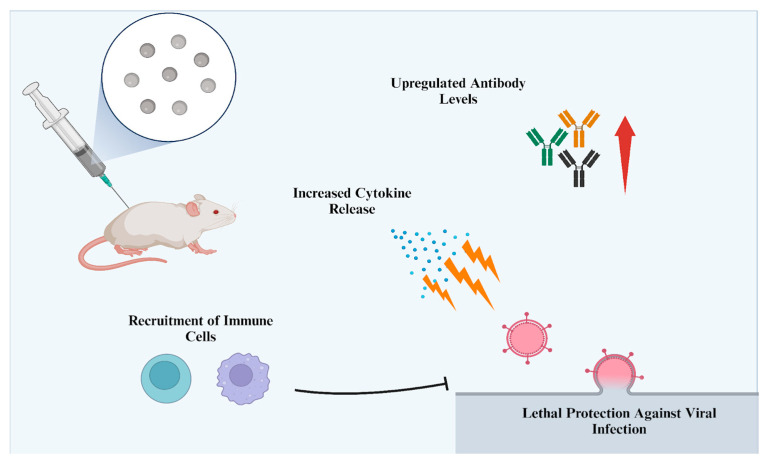
Usage of silver NPs as vaccine adjuvant for viral infections [147].

Since silver NPs can initiate the release of pro-inflammatory cytokines, induce inflammatory responses, and recruit certain immune cells, they are thought to be an alternative as a vaccine adjuvant [145]. Vaccine adjuvants are responsible for increasing the immune response against the targeted antigen, thus improving immunization during the process [146]. In this case, there are quite a few studies that show an increased immune response, in both *in vitro* and *in vivo* models, when silver NPs are used as an adjuvant (Figure 7).

As an example, it was demonstrated that the addition of silver NPs to a virus-inactivated flu vaccine reduced viral loads, prevented lung inflammation, enhanced immunoglobulin A-secreting plasma cells, and protected mice from lethal flu [147]. The effect of silver NPs on immune cells, antibodies, and inflammatory molecules was observed. It was found that silver NPs induced immune cell synthesis of neutrophils and monocytes and increased pro-inflammatory cytokines (IL-6 and IL-16) in mice in response to keyhole limpet hemocyanin (KLH) proteins. The same results were also observed in the lungs, inducing local inflammation, but only for the first three days. Additionally, silver NPs enhanced mucosal-specific immunity against KLH by inducing KLH-IgA and KLH-IgG antibodies, along with B-cells and IL-12 numbers. The authors indicated that these results showed that silver NPs induced immunity against KLH as an adjuvant in lung vaccination. Subsequently, the protection of mice from lethal flu by silver NPs was demonstrated. Heat-inactivated influenza A virus (HIAV) was administered to mice as an antigen, with and without silver NPs. The addition of silver NPs to the treatment induced the recruitment of B cells and T-helper cells, while the solo vaccine treatment barely showed any cell recruitment. The same results were also observed in B-cell-activating factors and IL-12 numbers. Most importantly, there was no trace of any IAV-IgA and IAV-IgG antibodies with only HIAV administration. The addition of silver NPs remarkably induced the titers of these antibodies (lung IAV-IgA levels were also induced). Certain types of cytokines (such as CCL-20 and CXCL-13) were significantly increased as well. Moreover, the addition of silver NPs prevented weight loss in mice and significantly reduced viral loads in the lungs, whereas HIAV administration could not. Finally, all mice from the silver NP-HIAV group survived the 18-day post-infection phase. Similar results were observed for the oral vaccination of an influenza DNA vaccine encapsulated in silver NPs in a chicken model [148]. The analysis of T-cell populations in chickens revealed that silver NP treatment significantly induced CD3/CD4 and CD3/CD8 cells at day 28. Changes in cytokine expression were greatest in the silver NP-encapsulated vaccine group, showing fold changes of between 2 and 18 after 14 days.

Other examples indicate the enhancement provided by the addition of silver NPs in other types of viruses and models. For example, it was emphasized that the addition of silver NPs to neutralizing antibodies decreased the infection of human immunodeficiency virus (HIV-1) *in vitro* [149]. Even though the combined treatment did not yield any additive results in cell-free HIV-1 virus, it managed to inhibit cell-associated HIV infection, while solely administered naturalizing antibodies could not. Another study included silver NPs in a vaccine formulation that targets *A. baumannii* in an *in vivo* experiment [51]. The additive vaccination significantly induced the IgG antibody response, prevented mice from lethal infection, and decreased tissue damage.

In summary, these results indicate that silver NPs possess significant potential to induce an immune response and cell recruitment following vaccination. The current literature provides well-established experiments that show the enhancement of many parameters while maintaining lethal protection against infections. Still, a detailed investigation of silver NP application as a vaccine adjuvant is lacking in current research. There could be different reasons behind the absence of studies: there might be better alternatives compared to silver NPs, the toxicity that comes with higher concentrations could be a drawback, or other applications might be preferred over vaccination applications. Still, there is a possibility that silver NPs can be administered in certain vaccines to enhance vaccination outcomes, especially considering their antimicrobial and inflammatory activities.

## 8. Diabetes Applications

**Figure 8:**
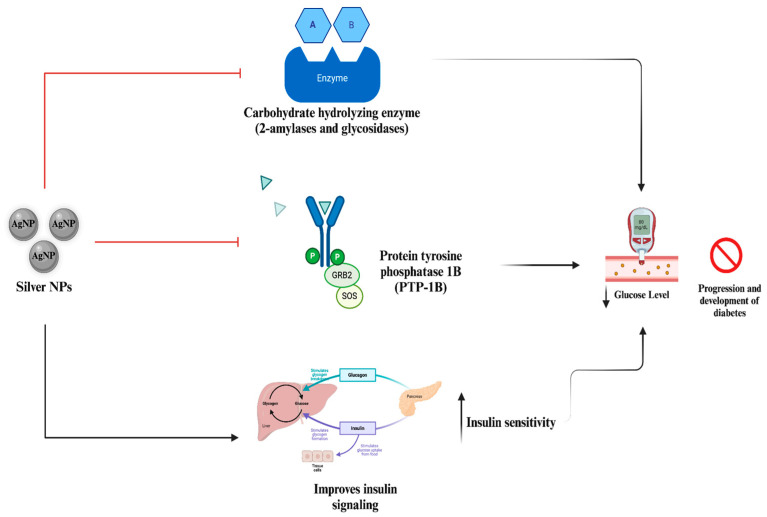
Antidiabetic activity of silver NPs [150].

Silver NPs are a good candidate for future antidiabetic applications since they possess certain major characteristics. For example, they exhibit enzymatic inhibition, which can cause a significant reduction in blood sugar levels, as discussed in the following studies (Figure 8) [150]. Additionally, they are applied in the treatment of diabetes-induced wounds due to their significant wound healing activity in diabetic conditions, along with their anti-inflammatory and antibacterial activities [151]. A meta-analysis using animal models also supported the future potential of silver NPs in anti-diabetic applications [152].

The antidiabetic activity of silver NPs was demonstrated in streptozotocin-induced diabetic mice [153]. During the experiments, certain parameters related to hyperglycemia were observed with silver NP treatment. At the highest concentration (100 μg/mL), silver NPs exhibited 78% radical scavenging capacity and managed to inhibit alpha-amylase at a rate of 83%. These two characteristics play an important role in the antidiabetic activity of silver NPs. Alpha-amylase is responsible for carbohydrate digestion and can strongly influence hyperglycemia [154]. On the other hand, diabetes-induced oxidative stress from increased amounts of free radicals is strongly associated with chronic hyperglycemia [155]. These results are thus extremely important for antidiabetic activity. Following these results, the blood glucose levels of mice were measured with and without silver NP administration. Both silver NP groups, with concentrations at 5 mg/kg and 10 mg/kg, showed significantly reduced blood glucose levels and improved glucose tolerance of diabetic mice. Finally, a change in gene expressions (*AMPK* and *IRS1*) was observed in the silver NP-treated groups. It was confirmed that both genes’ expression was significantly reduced in the diabetic groups compared to the control group. However, both silver NP groups had significantly increased gene expressions. Enzymatic inhibition and changes in blood glucose levels were also demonstrated in an *in vitro* study [156]. As the concentration of NPs rose, significant inhibition of alpha-amylase and alpha-glucosidase enzymes was observed by 52.48% and 55.6% at the highest concentration (1000 μg/mL), respectively. In addition, a glucose uptake assay showed that silver NPs inhibited glucose transport across the membrane by 73.33%. The authors concluded their research by highlighting the potential of silver NPs as a competitor in diabetes treatment and the requirement for further *in vivo* experiments.

A recent study demonstrated the biochemical characterization of streptozotocin-induced diabetic rats treated with green synthesized silver NPs [157]. Histological observations of the kidneys were conducted to assess the effect of streptozotocin-induced diabetes. Silver NP treatment showed kidney tissue restoration without inducing any cellular damage. Moreover, significant recovery of body weight and a reduction in glucose levels were also observed. Most importantly, several markers, such as cholesterol (HDL/LDL) and triglycerides, were improved in diabetes-induced rats. In addition to enzyme inhibition and alterations in blood glucose levels, silver NPs may also influence diabetes-based biochemical markers, indicating its potential as an anti-diabetic agent.

Apart from possessing direct activity against diabetes, silver NPs were also applied to reduce diabetes-based complexes. Alkhalaf *et al.* demonstrated the reduction of diabetic neuropathy by silver NPs through anti-inflammatory and antioxidant mechanisms in an *in vivo* rat model [158]. Similar to previous studies, silver NPs significantly decreased serum glucose and increased serum insulin levels in diabetic neuropathy groups. Additionally, there was a significant reduction in inflammatory markers, malondialdehyde, and nitric oxide levels, indicating a great reduction in oxidative status. Furthermore, there was a noteworthy increase in superoxide dismutase and glutathione levels. Related to its wound healing activity, silver NPs were also applied in the treatment of diabetes-induced wounds. For instance, the enhancement of diabetes-induced wound healing by silver NPs was demonstrated in diabetic rabbits [159]. Silver NPs were developed with a chitosan–polyethylene glycol hydrogel for the efficient release of the NPs. The antimicrobial, antioxidant, and wound healing activity of the silver NP hydrogel was tested during the study. Together with the histological analysis, the hydrogel successfully and efficiently healed diabetes-induced wounds.

It seems that many characteristics of silver NPs potentially influence their antidiabetic activity. They can regulate specific gene expressions, inhibit enzymatic activity, and modulate diabetic conditions by radical scavenging. Additionally, they can alleviate diabetes-based conditions by reducing proinflammatory molecules, enhancing wound healing, and influencing additional enzymes for inducing antioxidant mechanisms. The significant changes in glucose levels should not be unnoticed and studies involving animal models must be improved, considering toxicity conditions.

## 9. Dental Applications

Silver NPs have been included in various dental materials, such as acrylic resins, implants, porcelain restorations, composite resins, and so on [160]. These materials have been used for removable denture fabrication, to induce restoration, to inhibit pathogens in solutions, and for obturations [161]. The utilization of silver NPs in dental applications is extensive. This is why we evaluated some of the major materials that silver NPs have been included in.

The antimicrobial activity of silver NPs is highly preferred in dental materials. The management of potential oral biofilms is crucial in many dental applications, such as restorations, implants, and canal treatments [162]. Therefore, dental materials that can prevent biofilm formation are a necessity for the prevention of oral diseases. This is why silver NPs are a strong candidate for inclusion in dental materials, with their rich antimicrobial and antibiofilm characteristics.

The use of silver NPs in denture acrylic resin to inhibit biofilm formation was established [63]. A nanocomposite with poly (methyl methacrylate) (PMMA) resin and silver NPs was formed and tested against a *C. albicans* biofilm. The nanocomposite successfully decreased the number of cells in the biofilm while maintaining the material’s flexural strength. Additionally, there was no sign of an inflammatory response by the silver NPs after 60 days in the subcutaneous tissue of rats. Another study also used PMMA and added graphene–silver NPs to observe changes in the material’s properties, including the antimicrobial activity [163]. An inflammation test revealed that the addition of graphene–silver NPs decreased the inflammation that was induced by solo-administrated resin. Similar correlations were also observed in the antioxidant tests, but, this time, the effect of the silver NPs was significant compared to the control group. The antibacterial test, as expected, was highly significant among the tested properties. The material was extremely effective against *S. mutans*, *E. coli*, and *S. aureus*. Distinct from a previous study’s result, the flexural strength was significantly higher with graphene–silver NP-added resin, and graphene might have been the main reason for the increase.

Silver NPs are also included in acrylic resins with composite structures for similar reasons. A research-modified PMMA composite resin with cellulose nanocrystals was coated with polydopamine and silver NPs to enhance the material properties [164]. The composite showed an increase in its flexural strength by 12%, flexural modulus by 8.8%, and rupture work by 47.7% and 72.8%. The antibacterial activity was tested on *E. coli* and *S. aureus*. At a concentration of 0.10 wt.% or higher, both bacteria showed baseline concentrations, indicating the significant activity of the composite resin.

Silver NPs are also applied in dental implants and periodontal treatment due to their desired antibacterial activity. Hence, silver NPs hold great promise. Hernandez-Venegas *et al.* demonstrated the bactericidal activity of silver NPs on oral biofilms isolated from periodontal patients [165]. They observed that bacterial growth in periodontal patients was significantly higher compared to healthy patients, confirming the increased bacterial activity. This was further confirmed by a PCR assay showing an increased number of bacteria strains in biofilms. To observe the antibacterial activity, silver NPs were tested on biofilms from both periodontal and healthy patients. As expected, significant antibacterial activity was observed in all biofilms, especially those with smaller NPs.

De Giglio *et al.* showed the antibacterial activity on implants by silver ion release from synthesized silver NP-based titanium implant coatings [166]. The authors tested the antibacterial activity against *S. aureus*, *E. coli*, and *P. aeruginosa*, based on their involvement in orthopedics infections. Silver NPs managed to put bacterial growth levels at very low levels during the observed 30 h. Compared to the non-coated system, it was revealed that silver NPs were solely responsible for the antibacterial activity. In addition, silver NPs prevented biofilm formation during the experiments.

There are multiple types of dental materials that include silver NPs. As discussed, the primary reason for preferring silver NPs is their significant antibacterial activity. Bacterial growth and infections cause significant issues in dental applications. It can even lead to oral bacterial diseases that possess important risks. We discussed the involvement of silver NPs in dental resins and briefly mentioned their application in implants and periodontal disease to provide variety and avoid repetition. There are more types of dental materials where silver NPs are used, but it would be difficult to discuss all these materials. Therefore, we have included some of these materials in Table 1, along with additional examples of previously mentioned materials.

## 10. Anticancer Applications

**Figure 9:**
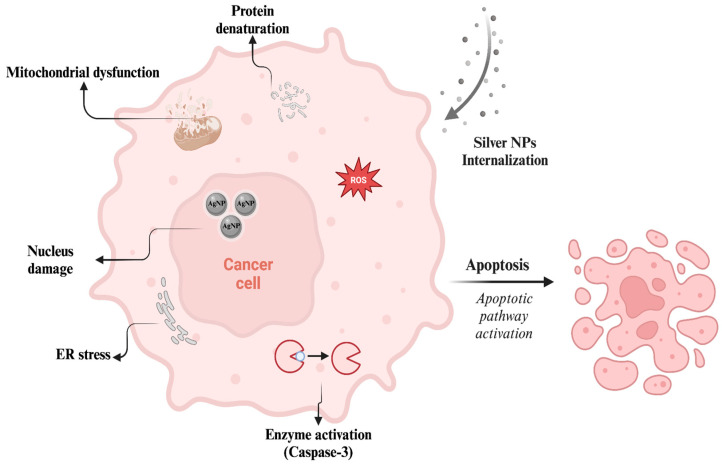
Anticancer mechanism of silver NPs [167,174].

Among the applications covered in this review, the anticancer activity of silver NPs is one of the most extensively studied features in current research. Silver NPs can utilize mechanisms that significantly induce the death of cancer cells. These particles can initiate anticancer activity by either inducing apoptosis through mitochondrial disruption and an imbalance of apoptotic protein levels or causing structural and functional damage to cellular components, which can also lead to apoptosis [167] (Figure 9). Additionally, it has been proposed that silver NPs can interfere with the cell cycle of cancer cells, potentially causing arrest in the sub-G1 phase [168]. The multiple mechanisms of silver NPs have led to their extensive application in anticancer research. Moreover, the anticancer efficacy of silver NPs can be greatly enhanced through strategies such as green synthesis from plants with anticancer properties and combined treatment with chemotherapeutic drugs [169]. These plant-based silver NPs can demonstrate anticancer mechanisms, such as increasing ROS levels, upregulating the tumor-suppressor gene p53, and activating caspase 3 for apoptosis induction [170]. These are the main mechanisms behind the anticancer activity of silver NPs, especially green synthesized ones, which have been predominantly utilized in anticancer research for at least five years. These mechanisms are featured in many studies, some of which mention apoptotic morphological changes and the expression of apoptotic proteins, along with ROS-mediated cell death [171,172]. Some studies also include the antiangiogenic activity of silver NPs [173].

In addition, the cell cycle arrest induced by silver NPs is widely investigated in anticancer research. The effect of silver NPs on human hepatocellular carcinoma HepG2 cells was demonstrated for potential application in liver cancer treatment [175]. The initial factors influenced by silver NP treatment were increased nitrite production and ROS generation. Subsequently, it was found that increased nitric oxide (NO) and ROS levels caused the depolarization of mitochondrial membrane potential and cell cycle arrest (significant in the G2/M phase) mediated by DNA damage. A study demonstrated that medical plant biosynthesized silver NPs exhibit cytotoxicity by interfering with the cell cycle [176]. The anticancer activity was tested on A375 skin melanoma cells, along with the antibacterial activity on *E. coli*. The silver NP-treated melanoma cells showed DNA ladders in the DNA fragmentation study, indicating apoptosis. Afterwards, a cell cycle analysis was performed to determine the main mechanism behind silver NP-induced apoptosis. At the highest concentration of silver NP treatment, a significant arrest in the G2/M phase of cells was observed, reaching 50.8%. A similar cell cycle arrest mechanism was demonstrated in human prostate (DU145) cancer cells [177]. Green synthesized silver NPs dose-dependently caused cellular death in DU145 cells. In the first 24 h of treatment, a 24% decrease in cell numbers and 5.8% cell death were observed. After 48 h of the treatment, these percentages increased to approximately 1.5-fold and 3-fold of the previous values, respectively. In both timelines, higher doses of silver NPs caused G2/M cell cycle arrest, while lower doses caused G1 phase arrest.

The physical properties of silver NPs should also be considered when evaluating their anticancer effects. Pucelik *et al.* highlighted the importance of silver NP size. Evaluating NPs with varying sizes from 3 to 40 nm, they revealed the greatest efficiency with 40 nm silver NPs. These larger particles demonstrated strong anticancer effects against murine colon carcinoma (CT26) and murine mammary gland carcinoma (4T1) cells while minimizing harmful effects towards human HaCaT keratinocytes [178].

It is important to note that the toxicity induced by silver NPs and silver ions has significant potential in anticancer studies, as it can prevent cell proliferation, induce DNA damage and oxidative stress, and disrupt cellular membranes [179]. Additionally, we have briefly mentioned that the physical properties of silver NPs alter their toxicity. These properties play an essential role in the cellular uptake of silver NPs and the induction of ion release, thereby initiating anticancer mechanisms. Therefore, it is crucial to determine the properties of silver NPs during synthesis to achieve the desired level of toxicity for applications.

## 11. Biosensor and Bioimaging Applications

Silver NPs have distinct optical properties, leading to their utilization in biosensor and bioimaging applications. They possess potential for the bioimaging of certain proteins, DNA, and tumor cells due to their plasmon resonance and surface modifiability [180]. Additionally, silver NP-conjugated probes exhibit great sensitivity to many biomolecules, especially DNA and antibodies, and are used for their detection in several sensors [181]. They exhibit extreme efficiency in LSPR excitation, an enhanced wavelength range, sharper bands, and a greater refractive index, enhancing their usage in biosensor applications [182].

The optical properties of silver NPs, especially LSPR properties, are heavily influenced by their physicochemical properties. Multiple types of silver NPs (such as triangles, nanobars, and nanorods) of various sizes (usually up to 100 nm) have been tested for SERS-based sensors [183].

The size of silver NPs is directly proportional to the LSPR peak positions (nm), as an increased size can cause a red-shift in LSPR peaks [184]. Kravets *et al.* used silver NPs as imaging agents for rat basophilic leukemia cells and neural stem cells. A red shift was reported in both photoluminescence emission and resonance extinction as the particle size increased from 9 nm to 32 nm, underlining the role of SPR in bioimaging [185].

The influence of size on detection efficiency was evaluated by Fuke *et al.*, who developed a polyaniline–silver nanocomposite for humidity detection. By comparing the performance of 15 nm and 30 nm silver NP-incorporated sensors, they found that smaller silver NPs considerably improved the sensitivity and were able to detect relative humanity across a wide range, from 5% to 95%. This enhancement could also be correlated with LSPR, as 15 nm silver NPs showed decreased absorption peaks (at 400 nm) in comparison with the 30 nm NPs (420 nm) [186]. 

The structure of silver NPs similarly influences LSPR peak regions. Several studies have recorded different LSPR peaks for various silver NP structures. One study highlighted that triangular silver NPs showed an LSPR peak at 579 nm, which red-shifted to 599 nm after gold coating [187]. Another study observed an LSPR peak for silver nanostars at ∼375 nm, which shifted up to ∼450 nm depending on the gold concentration added [188]. The LSPR peaks of silver nanospheres are highly size-dependent, with peaks around (mean) 410 nm [189]. A dominant LSPR peak at 495 nm was demonstrated for silver nanocubes [190]. These studies clearly indicate variation in the optical properties of silver NPs based on their structure. These peaks influence the efficiency and sensitivity of silver NP-based biosensors. Therefore, the physicochemical characteristics of silver NPs must be determined to obtain the desired LSPR peaks for optimal biosensor development.

The enhancement of LSPR optical sensors by silver NPs has been demonstrated [191]. Two parameters were evaluated to increase the sensitivity of a sensor for biosensing. First, a significant increase in refractive index sensitivity was observed when the sensing length was higher (275 nm/RIU to 379 nm/RIU). The reason behind the increased sensitivity was thought to be the increased number of immobilized silver NPs on the sensing surface. Secondly, the increased coating time of silver NPs significantly increased sensitivity, from 173 nm/RIU to 461 nm/RIU. However, when the coating time reached 3 h, the sensitivity decreased to 355 nm/RIU, potentially due to increased aggregation, indicating that the optimum coating time was 1 h. The enhanced silver NP-based optical sensor was tested on the antigen–antibody interaction (human IgG and rabbit anti-human IgG). The antigen–antibody binding was monitored through the progressive red-shift of the LSPR peak wavelength.

Loiseau *et al*. demonstrated the use of two different NPs, gold–silver (core) shell NPs and silver–gold (core) shell NPs, for the LSPR-based naked-eye biosensing of staphylococcal enterotoxin A [192]. It was stated that increased silver concentrations enhanced refractive index sensitivity. The LSPR bands of both NPs red-shifted and led to visual color changes. Additionally, the attachment of anti-SEA antibodies to gold–silver (core) shell NPs could be observed by the naked eye through a clear color change from orange to red. The color change was less observable for silver–gold (core) shell NPs, as the colors changed from orange to deep orange/brownish. It was concluded that both NPs yielded high performance based on their limits of detection.

Silver NP-containing SERS-based sensors provide a different approach for agricultural applications. A new study showed the sensitive detection of foodborne pathogens on an egg surface with SERS-based sensors, where silver nanocubes assembled on polydimethylsiloxane membranes [193]. Both *E. coli* and *Salmonella typhimurium* were added in random concentrations in egg samples. The system showed a more than 93% recovery rate, indicating its promise for application to real samples.

Since silver NPs can effectively penetrate cells due to their small size and be subjected to surface modifications, they are highlighted candidates for bioimaging studies [194]. The cellular penetration and intracellular activity of silver NPs are prominently highlighted in anticancer studies. In this context, cancer cells are ideal targets for silver NP-based bioimaging applications. This was evaluated in a study involving biosynthesized silver NPs for cancer therapy and *in vitro* and *in vivo* bioimaging [195]. *In vitro* fluorescence studies in both normal and cancer cells showed that silver NPs entered all the cell lines and exhibited significant red fluorescence. The difference between the normal cells and cancer cells revealed the ongoing apoptotic status of the cancer cells. Later on, *in vivo*, near-infrared (NIR)-based fluorescence was used on silver NPs and demonstrated bioimaging at 710 nm excitation and 820 nm emission. The bioimaging results showed the accumulation of silver NPs in the brain, along with certain vital organs, indicating the penetration of the NPs. Ex vivo imaging revealed that silver NPs accumulated highly in the brain, kidney, colon, liver, and lung. Bioimaging of rat basophilic leukemia and neural stem cells was also demonstrated by surface modification of NPs with fluorescent glycine dimers [185]. Similarly, the smallest silver NPs were chosen for efficient cellular penetration. Confocal microscopy images of cell types showed visible results confirming the accumulation of silver NPs in the cellular membrane.

Silver NPs can be utilized in many bioimaging and biosensor applications. Their small size and well-known cellular penetration ability make them preferable materials. Most importantly, the surface modifiability of silver NPs widens the range of their usability in these applications. They can be functionalized with cell-targeting biomolecules to obtain enhanced images and monitor molecular interactions.

## 12. Toxicity

Silver NPs are highly advantageous and should be considered for innovative and difficult biomedical applications; yet, only recently has their toxicity been the focus of extensive research. Humans typically ingest about 0.4 to 30 µg of silver daily from natural sources in food and water [196]. Studies that have been conducted on the adverse effects that silver NPs can have on biological systems, including human cells, bacteria, and viruses, have produced inconsistent findings [197]. The prevailing consensus is that silver NPs are extremely potent antibacterial agents that are not detrimental to healthy mammalian cell cultures [198]. Nonetheless, a number of *in vitro* investigations have shown nanosilver-induced toxicity in human lung epithelial cells [199], murine stem cells, rat hepatocytes, and neural cells [200].

ROS induction is the main mechanism by which silver NP-dependent cytotoxicity in *in vitro* experiments is achieved. The key determinants of silver NP cytotoxicity and genotoxicity are their size, concentration, and exposure time [201]. Due to their small size, silver NPs can cause toxicity by piercing biological membranes and entering cells. The effect of particle size on the toxicity capacity of silver NPs has been demonstrated [202]. The size of the tested NPs varied from 20 nm to 100 nm. The zebrafish model revealed that small-size NPs demonstrated the highest mortality rates, along with the higher concentrations. On the contrary, the largest silver NPs, 100 nm, did not show mortality higher than 20%, even at the highest concentration, which was 12 mg/L. The adverse effects on the model were significant with the 20 nm and 40 nm sizes, while the 80 and 100 nm sizes barely showed any.

**Figure 10:**
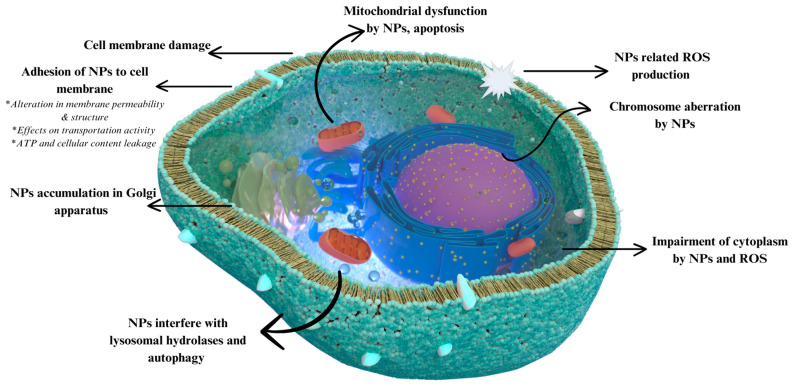
Toxicity mechanisms of silver NPs [211].

The toxicity is mostly linked to the production of ROS, which causes oxidative stress, cellular malfunction, and inflammation in a variety of tissues, even if the precise process is yet unknown. This process interferes with how cells work and can have a negative impact on health by causing organ failure, DNA damage, and the emergence of chronic illnesses (Figure 10) [203]. Also, glutathione levels are lowered, lipid peroxidation occurs, ROS-responsive genes are expressed more, and there is an increase in their protein levels because of silver NP exposure. These events ultimately result in DNA damage, apoptosis, and necrosis [201]. In more detail, Patlolla *et al.* evaluated the *in vivo* toxicity of orally administered silver NPs in a rat model. Their findings revealed that exposure to varying doses of silver NPs, 5, 25, 50, and 100 mg/kg, increased the generation of ROS, activities of liver enzymes, and lipid hydroperoxide levels and led to morphological changes in rat liver tissue. They also noted a marked increase in these adverse effects, especially at the highest doses, 50 and 100 mg/kg [203].

Similarly, Kim *et al.* focused on demonstrating the role of oxidative stress on the toxic effects of silver NPs in human normal bronchial epithelial cells (BEAS-2B). Utilizing *in vitro* tests, including comet and micronucleus (MN) assays, they observed a notable increase in oxidative DNA damage caused by silver NPs through the generation of ROS. Furthermore, their findings highlighted that silver NPs stimulated DNA breakage and MN formation in BEAS-2B cells in a dose-dependent manner [204].

Apart from showcasing the advantageous characteristics of silver NPs, such as their antibacterial and antifungal activity, *in vitro* investigations have also exposed the harmful and detrimental impact of silver NPs on bacteria or cells. A study demonstrated the toxicity of silver NPs in various biological systems [205]. Certain strains of viruses, fungi, bacteria, protists, and mammalian cells (including cancer cells) were treated with silver NPs. In all biological systems, silver NPs exhibited toxicity at certain concentrations. All tested animal cells and cancer cells showed zero cell viability at a 10 μg/mL concentration. Virus and bacteria strains possessed similar effective/MIC values at 12 μg/mL. The MIC values were higher in fungi (20 and 45 μg/mL) and lower in protists (4 μg/mL). It was highlighted that concentrations near 10 μg/mL could induce toxicity in multiple biological systems *in vitro*.

In toxicology research, different chemical doses are applied to cells and organs and the responses are tracked over time. These dose-dependent reactions aid in establishing proper dosages of the medication, exposure thresholds to prevent adverse effects, median toxicity (MD_50_), and median lethal dose (LD_50_) [206]. The focus of conventional cytotoxic assays is primarily on soluble substances that exhibit cellular toxicity following injection. When it comes to NPs, this is established based on dimensions, forms, and densities. This causes the NPs to diffuse across membranes and aggregate and agglomerate at locations in the target cells or organs, producing a colorimetric result. As a result, the results of conventional *in vitro* tests using NPs are less trustworthy since the cellular absorption data are misinterpreted [207].

The effect of dose concentration on silver NP toxicity was demonstrated in a dose-dependent study on a *Drosophila* model [208]. The egg-laying capability was significantly reduced at the highest concentrations, 50 mg/L and 250 mg/L. In both lower and higher concentrations, the fertility of the Drosophila was not significantly affected on days 3 and 10. Additionally, lower concentrations did not significantly change the number of eggs laid, even by day 30.

A study showed the distribution of silver ions from silver NPs in rats [209]. Histological and genotoxicity studies showed that silver ions accumulated primarily in the lung, secondarily in the spleen, and thirdly in the liver. Subsequent accumulation in the kidney, thymus, and heart was also shown, respectively in order of detection. Alterations in cell structure and chromosome aberrations were shown to be the cause of the toxicity of silver ions.

The potential causes of silver NP toxicity from *in vivo* studies are far less well understood than from *in vitro* investigations. The subject of whether silver NPs truly a hazardous effect on a wide range of species have, including terrestrial invertebrates, vertebrates, aquatic organisms, and higher plants, is addressed by *in vivo* studies on the cytotoxicity and genotoxicity of silver NPs. Therefore, through routes including ingestion, skin contact, and inhalation, organisms are readily exposed to NPs. Silver NPs have the ability to infiltrate cells and migrate to other important organs [207]. After local injection of silver NPs, exposure to silver NPs has resulted in a multitude of toxicological reactions, including effects on the liver, skin, central nervous system, circulatory system, and respiratory system [210].

## 13. Industrial Applications

**Figure 11:**
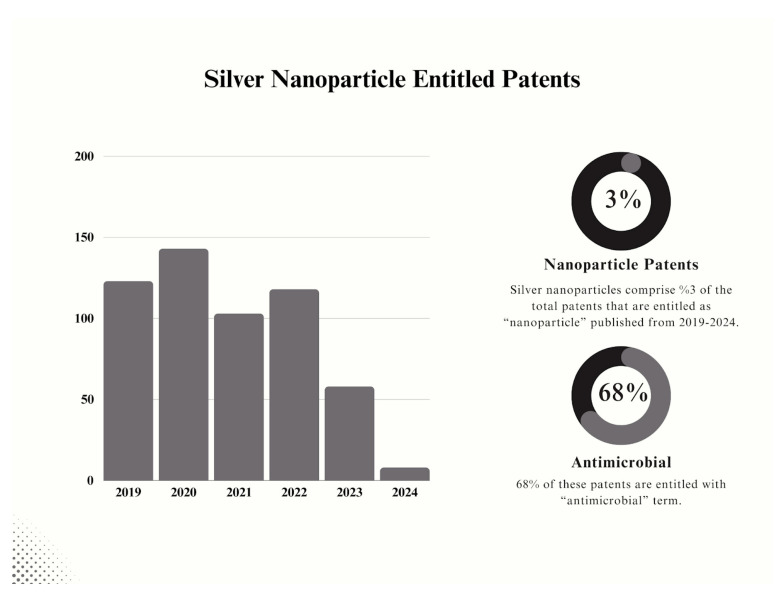
Graph representing the published patents that include the term “Silver Nanoparticles” in their title between 2019 and 2024 [214].

The industrial applications of NPs extend to wide-ranging areas, including but not limited to the chemical industry, for uses in cosmetics, color filters, and catalysts; as antimicrobial additives in packaging; and as biomaterials used in, for example, bone and tissue engineering [212]. Since silver exhibits strong characteristics, some areas possess a high potential for the utilization of silver compared to other application areas. For instance, as mentioned in this review, the antimicrobial activity of silver is predominantly found in most silver NP applications. A significant increase in registered patent numbers for antimicrobial applications of silver can be observed from 2013 [213]. For the last five years, there have been more than 500 patents entitled “silver nanoparticles” in Google patents, indicating the potential of silver NPs in the future of industrial applications [214] (Figure 11). In total, 68% of these patents involve the term “antimicrobial” in their title, supporting the dominance of the antimicrobial activity of silver NPs in the industry. Even though the approximate results show that the number of patents using the term “silver nanoparticle” in their title only comprises 3% of the total patent numbers published between 2019 and 2024, this ratio is quite acceptable when the extremely wide-ranging applications of silver NPs are considered. Except for the last year, excluding this year, there have been more than 100 patent registrations based on silver NPs. In 2023, the peak of patent registration nearly dropped by half compared to 2022. There has been a more significant reduction in 2024, indicating a clear decline. The observable reduction in the last few years might show a shift in research on innovative approaches. This is quite possible considering the number of silver NP research papers over the years.

The antimicrobial properties and mechanisms of silver NPs are well understood, as we have discussed. Considering the need for alternative agents to antibiotics as bacteria develop resistance to commercial drugs, it is reasonable that there is extensive research on the antimicrobial activity of silver NPs. Additionally, the ability to incorporate the antimicrobial activity of silver NPs in, for example, wound dressings, food packaging, and medical devices, is an additional factor driving the predominant research on bacteria. Interestingly, given the shared potential of the antibacterial field and environmental safety in degrading organic pollutants (especially dyes), many recent studies have utilized the antimicrobial activity of silver NPs in dye degradation [215,216]. Catalytic reduction mediated by silver NPs shows high potential in wastewater treatment, providing another example of antimicrobial activity being applied in different fields. This was further highlighted in recent research demonstrating the potential use of silver NPs in marine antifouling with both antibacterial and anti-algal activity [217].

However, it is important to highlight that using NPs, especially silver NPs, as an alternative to antibiotics requires critical investigation into their long-term effects on the ecosystem, particularly in a scenario where silver NPs could replace antibiotics in certain areas [218]. Considering this possibility, a new research area focusing on the effects of excessive amounts of silver NPs on the environment could potentially become a major topic in the near future. Given the predominant research on green synthesis, the next step in the development of silver NPs in the industrial sector may focus on environmental studies.

Similar factors apply to the anticancer applications of silver NPs, as cancer research is in high demand due to cancer being one of the most critical health challenges. One of the most beneficial aspects of silver NPs’ toxicity is their significant potential in anticancer studies. They possess multiple mechanisms, have the characteristics to easily initiate cellular entry, and can be modified for precise cell targeting. Their potential as nanocarriers is significant, given the need for natural compound-based nanocarriers to enhance targeted drug delivery [219]. As both carriers and potential anticancer agents, it is unsurprising that silver NPs are heavily investigated for their anticancer properties in the current literature.

Despite the wide-ranging sub-areas in agriculture where silver NPs can be applied, such as food packaging, nanofertilizers, and nanopesticides, their industrial applications in agriculture remain quite limited. Even though silver NPs account for 18.6% of studies in the last 5 years, most of these studies involve their antibacterial activity. The primary reason for the limited industrial application of silver NPs in agriculture is the high potential for environmental damage that they may cause. The excessive use of silver NPs in plants, soils, and water ecosystems is still not fully understood. It is clear that this slow research progress significantly hinders the industrial development of silver NPs in agriculture.

A similar hindrance is also observed in the antiviral research on silver NPs. As mentioned, silver NPs can exhibit both antiviral activity and function as adjuvants in vaccines. The uncertainties regarding the effects of silver NPs on humans, their potential toxicity in *in vivo* models, and the gaps in the understanding of their antiviral mechanisms also slow progress in this area.

Studies on wound healing applications have comprised a significant portion of research in recent years. We have shown that silver NPs are highly effective in wound healing applications, primarily in *in vitro* experiments, with limited success in mice in *in vivo* studies. Silver NPs are also effective in bone healing studies, but there have been very few recent studies in this area. Similar limitations, such as biocompatibility and toxicity concerns, also slow research on silver NP-based wound healing and bone repair, compared to applications like antimicrobial studies.

## 14. Limitations and Challenges of Silver NP Applications

Silver NPs exhibit a wide-ranging area of applications with significant potential and efficiency. Still, this variety carries some current limitations and future challenges on the road to achieving the full potential and deployment of silver NPs in these discussed areas.

### 14.1. Toxicity Potential

The toxicity potential of silver NPs could be the primary challenge and limitation hindering their development in many fields. Silver NPs exhibit significant toxicity potential for various plants, aquatic animals, and, most importantly, humans [220]. We have mentioned several areas with potential for future human applications and discussed agricultural uses that directly involve many plants. Depending on their properties and concentration, silver NPs pose a significant risk to both plants and humans during application. Given their toxicity mechanisms, exposure to high concentrations of silver ions can induce severe cytotoxicity through oxidative stress and apoptosis. In terms of plant-based applications, the experimental approach in silver NP-based agriculture applications influences the toxicity capacity as much as the physicochemical properties of the particle [221]. The concentration, amount of time that plants will be exposed to particles, and physiology of the plant can significantly alter the toxicological effects of silver NPs. Based on silver NPs’ toxicity mechanisms, they could be involved in various types of toxicity in humans, such as cardiovascular diseases, immunotoxicity, and neurotoxicity [222]. Additionally, the risk of toxicity in aquatic environments is a major concern, as many aquatic plants and organisms are highly susceptible to high concentrations of silver ions [223]. Silver NPs exhibit high toxicity potential for many zooplankton species, leading to oxidative stress, reproductive inhibition, severe DNA damage, and high mortality rates [224]. The physicochemical properties of silver NPs strongly influence their toxicity potential. The shape of silver NPs influences their affinity for molecular interactions, increasing the potential for unwanted interactions in biological systems and aggregation during treatment. Small-sized particles show high cytotoxicity and the potential to enter other parts of biological systems. The surface composition and charge of silver NPs are similarly important in this context. This highlights another key challenge and limitation of silver NP applications: gaps in optimization within the current field.

The significance of toxicity potential is well documented in the current literature. In recent years, studies involving silver NPs have increasingly used green synthesis methods to overcome the many disadvantages of NP applications, particularly toxicity [225]. With lower energy demand, eco-friendly reactions, and more reliable, less complicated procedures, green synthesis of silver NPs promises near-non-toxic levels of toxicity [226]. As a result, many studies have investigated the activity, stability, and, particularly, toxicity of novel synthesized silver NPs from various sources, such as plants, microorganisms, and bioactive compounds. Innovative developments in green synthesis studies could be the starting point for eliminating toxicity in current silver NP challenges.

### 14.2. Gaps in the Optimization

As briefly discussed in this review, the physicochemical properties of silver NPs significantly influence both their applications and toxicity potential. The current literature discusses a wide range of shapes and sizes of silver NPs across various applications. However, no comprehensive standards exist for the shapes and sizes of silver NPs in these areas. These properties can heavily influence the efficiency of silver NPs. Most importantly, their toxicity risk is also shaped by these properties. The absence of accepted standards for silver NPs in the current literature significantly hinders their commercial development. Optimizing silver NP standards is not only necessary for current industrial applications but also crucial for addressing future challenges, such as ethical concerns.

### 14.3. Future Resistance to Silver NPs

Given the significant antibacterial activity of silver NPs, the predominant research focus in the current literature is unsurprising. We have discussed the potential of silver NPs to overcome antibiotic resistance and their effective toxicity against multi-drug-resistant bacterial strains. However, the possibility that bacteria susceptible to silver NPs could develop resistance, similar to antibiotics, is a major concern that must be addressed in current applications [227]. The physicochemical properties of silver NPs must be carefully controlled through synthesis methods, and the dose of administered silver NPs should be optimized to prevent bacteria from developing resistance mechanisms. Bacteria can possess various mechanisms against silver NPs that can lead to the agglomeration of particles, inhibition of dissolution, prevention of particle interaction, expulsion of silver ions from cells, and so on [228]. Methodologies that can help overcome resistance mechanisms, such as surface modifications and co-administration approaches, should be further investigated. Still, it needs to be remembered that resistance mechanisms can show variances according to the size and surface modification of silver NPs [229].

### 14.4. Stability and Degradation of Silver NPs during Treatments

Surface modifications of silver NPs are not only important for overcoming resistance mechanisms but also effective in reducing potential toxicity and ensuring stability to prevent agglomeration during application. Depending on environmental conditions, silver NPs may release varying amounts of silver ions, which can significantly affect the efficiency of the application. Additionally, it needs to be highlighted that silver NP-based bioimaging and biosensing studies are highly sensitive to agglomeration. The agglomeration of silver NPs involves large particles and negatively influences the stability and efficiency of the particles [230]. Achieving high stabilization of silver NPs through optimal environmental conditions and surface properties can also help ensure storage quality for both the NPs and the nanomaterials containing silver NPs.

### 14.5. Manufacturing and Cost Challenges

The broad application of silver NPs poses a significant challenge in optimizing cost-effective manufacturing. Since the physicochemical properties of silver NPs are heavily influenced by the synthesis process, controlling the size and shape of silver NPs in large-scale production is a major limitation. It can be assumed that clinical uses of silver NPs are heavily hindered by the lack of consistent procedures for large-scale productions and the incapability to predict the results of therapeutic applications [231]. This challenge is also addressed by green synthesis approaches. Additionally, surface modifications of silver NPs are sometimes essential to ensure safety and maintain efficiency in applications. Using this approach, the large-scale functionalization of silver NPs can be extremely challenging.

### 14.6. Potential Molecular Interactions of Silver NPs in Biological Systems

Regarding the future of silver NPs in human research, their distribution within the human body remains poorly understood. We are aware of the potential toxicities that silver NPs can cause due to their accumulation in specific organs, such as the liver and lungs. However, the potential side effects of this exposure and the body’s mechanisms for removing excess silver NPs are not fully understood. As mentioned in the previous sub-section, the surface modification and chemistry of silver NPs can significantly impact the interaction of the particles with biological systems [232]. Additionally, the potential interactions of silver NPs with various cells [233] and proteins [234] needs to be studied in biological systems to better predict undesirable outcomes.

## 15. Conclusions and Future Trends

Silver ions are one of the most efficient antimicrobial agents that are utilized in current nanotechnology research. They possess extremely important physicochemical characteristics and have wide-ranging applications. They are significantly effective in antimicrobial and antibacterial applications. They exhibit significant efficiency in interacting with cellular membranes and influencing intracellular parameters. These attributes are valuable in anticancer, antimicrobial, and bioimaging applications, along with agricultural, dental, and wound healing applications, since their antimicrobial activity is their most highlighted trait in these areas. 

Also, when the findings are considered, silver NPs can affect different variables, contributing to their application potential. They positively affect the physical properties of dental materials, food packaging, and bone scaffolds. In addition, they influence many characteristics of plants such as seed germination, plant length, and plant weight when administered as a nanofertilizer. When sufficient data are acquired in the future, these enhancements can prevent the potential development of health abnormalities. Moreover, they can act as radical scavengers, have a two-edged inflammatory influence, and alter enzyme activity in some key applications. Each of these activities shows the significant potential of NPs in the biomedical area. The antimicrobial activity of silver NPs will most probably be utilized in this field. Considering the extensive research published in the current literature, silver NPs might be more frequently included in studies on antibiotics and multi-drug-resistant bacteria in the future. There is a large body of research on wound healing applications. An innovative perspective could enhance the incorporation of silver NPs with biomolecules and their compatibility as scaffolds, thereby advancing silver NP-based regenerative studies. It is important not to underestimate the significance of the physical properties of silver NPs. Although there has been a considerable number of biosensor applications studied in the last few years, bioimaging studies make up a smaller percentage in terms of published papers. The compatibility of silver NPs as sensing materials and for bioimaging systems can be expected to be enhanced, particularly with the current ongoing patent registrations. The sustained volume of silver NP research clearly indicates the high demand for these particles in the market. The increase in the industrial applications of silver NPs will clearly amplify the need for silver NPs in many areas.

However, the toxicity potential hinders the flexibility of silver NP applications. Even though higher concentrations yield positive results in certain applications, such as anticancer and some antimicrobial applications, most of the time, they lead to negative outcomes. Their toxicity is mostly determined by the concentration and size of the silver NPs. Even though increased concentrations enhance silver NP activity, exceeding certain thresholds can lead to negative results. The area, size, and concentration parameters should be carefully determined to preserve the efficiency of silver NPs in biomedical applications.

## Figures and Tables

**Table 1:** Recent silver nanoparticle applications in various biomedical areas.

Application	Source of NPs	Properties	Results	Reference
Antibacterial	Green synthesis using *Brassica vulgaris* (*B. vulgaris*), *Brassica nigra* (*B. nigra*), *Capsella bursa-pastoris* (*C. bursa-pastoris*), *Lavandula angustifolia* (*L. angustifolia*), and *Origanum vulgare* (*O. vulgare*).	Size = 800, 912, 820, 40, and 46 nm average diameter for silver NPs synthesized from *B. vulgaris*, *B. nigra*, *C. bursa-pastoris*, *L. angustifolia*, and *O. vulgare*, respectively. Shape = spherical and truncated octahedron for smaller and larger NPs, respectively	-Significant antibacterial activity by five different types of green synthesized silver NPs, indicated by relative inhibition zone diameter ratio.	[13]
Antibacterial	Green synthesis using *Zataria multiflora*	Size = average hydrodynamic diameter of 25.5 nm Shape = spherical	-Stronger antibacterial activity and biofilm inhibition by plant-mediated silver NPs compared to commercial counterparts, with minimum inhibitory concentration (MIC) values of 4 µg/mL and 8 µg/mL against *Staphylococcus aureus* (*S. aureus*).	[14]
Antibacterial	Green synthesis using *Lawsonia inermis* (henna) leaves	Size = average diameter of 3.48 to 19.34 nm Shape = spherical	-Significant antibacterial activity of green synthesized silver NPs against multiple resistant bacterial strains.	[15]
Antibacterial	Green synthesis using Energy Cane Bagasse Hydrolysate	Size = approximately 15 nm Shape = spherical	-Significant antibacterial activity against both Gram-positive and Gram-negative bacteria with cellular attachment of the silver NPs. -Prevention of biofilm formation up to 100% at the highest concentration (250 µg/mL).	[16]
Antibacterial	Green synthesis using *Enterococcus faecium*-derived exopolysaccharides.	Shape = predominantly quasi-spherical structure SPR absorbance = 456 nm	-Significant antibacterial activity against both Gram-positive and Gram-negative bacteria. -Antioxidant activity in DPPH test.	[17]
Antibacterial	Green synthesis using aqueous extract of *A. australe*	Size = 15 ± 3 nm Shape = spherical SPR absorbance = 411 nm	-Significant antimicrobial activity on various bacteria, yeasts, and dermatophytes.	[18]
Antibacterial/Antibiotic	NPs synthesized using silver nitrate (AgNO_3_) and D-glucose as the reducing agent	Size = ∼26 nm Shape = spherical	-Inhibition of all bacterial strains with solo administration of silver NPs at a concentration of 10 μg/mL. -Demonstration of combined treatment leading to increased susceptibility and nearly complete inhibition of most resistant bacteria compared to solo treatments.	[19]
Antibacterial/Antibiotic	NPs were synthesized using chemical reduction and precipitation processes	Concentration of silver NPs was determined to be 1.7 µg/mL	-Enhancement of the activity of antibiotics against multidrug-resistant bacteria isolated from burn wound infections. -Increased activity determined by a 5.5- to 8-fold increase in sensitivity of isolates. -Enhanced *in vivo* healing activity and wound contraction observed with a spray formulation of silver NPs and neomycin antibiotics.	[20]
Antibacterial/Antibiotic	Stock silver NPs, with a concentration of 20 µg/mL, were purchased commercially	Size = 10 and 20 nm Shape= spherical	-Synergistic antibacterial activity between silver NPs and various antibiotics. -The MIC values were dropped by ½ or ¼ by the involvement of silver NPs.	[21]
Antiviral	NPs were synthesized using AgNO_3_ and NaBH_4_ as reducing agents, with TSC (thiosemicarbazide) acting as a capping agent	Size = the synthesized silver NPs had average sizes ranging from 6.2 ± 2.6 nm to 13.4 ± 4.0 nm, using different synthesis parameters such as dropwise addition of reagents and varying concentrations of silver precursor and reducing agents Shape = spherical	-Approximately 100% reduction in severe acute respiratory syndrome coronavirus 2 (SARS-CoV-2) viral load after two hours of infection.	[22]
Antiviral	Green synthesis using *Spirulina platensis* (*S. platensis*) and *Nostoc linckia* (*N. linckia*)	Size = average sizes of 21.211 and 21.052 nm for *S. platensis* and *N. linckia*, respectively. Shape = spherical	-Significant inhibition of hepatitis C virus (HCV) by 64.9% compared to ribavirin at 66.6%.	[23]
Antiviral	Green synthesis using *Punica granatum* (pomegranate) peel extract	Size = average size of 33.37 ± 12.7 nm Shape = spherical or round-shaped	-Significant increase in root and shoot length and plant weight in TMV-infected tomato plants treated with green synthesized silver NPs. -Significant decrease in TMV coat-proteins in all phases of treatment (before/after infection and dual treatment); 3-fold and 5.48-fold inductions in the expression of PR-1 genes, depending on the treatment phase (highest in the dual treatment). -Silver NP treatment showed a 50% decrease in the induction of PR-2 gene expression by TMV infection.	[24]
Antiviral	Green synthesis using *Rhizobium leguminosarum*	Size = ranging between 13.7 and 40 nm. Shape = spherical	-Significant restoration of weight loss and increase in chlorophyll concentration, along with a decrease in total protein, through administration of silver NPs before bean yellow mosaic virus infection. -Treatment showed a 3.34-fold increase in PR-1 gene expression and 2.31-fold increase in hydroxycinnamoyl transferase expression. -Reduction in oxidative stress markers and lower antioxidant enzymes compared to untreated infected plants.	[25]
Antiviral	Chemical synthesis	Size = approximately 7–8 nm SPR absorbance = 400 nm Shape = spherical	-Direct interaction of chitosan-modified silver NPs with swine coronavirus virions (12% viral attachment reduction). -Significant inhibition of viral replication.	[26]
Antifungal	Green synthesis using *Bacillus thuringiensis* MAE 6	Size = average size of 32.7 nm Shape = Spherical	-Antifungal activity against several *Aspergillus* spp., supported with inhibition zones of 16–26 mm at 500 μg/mL and MIC values between 15.62 and 125 µg/mL.	[27]
Antifungal	Green synthesis using Beech bark extract	Size = medium size of 32 nm Shape = spherical or sometimes triangular and polygonal	-Antifungal activity of biosynthesized silver NPs against *Candida* spp., with low MIC values, reflected by growth rates.	[28]
Antifungal	Stock silver NPs with a concentration of 5 mg/mL were purchased commercially	Size = average size of 5 nm Shape = spherical	-Inhibition of four types of kiwifruit rot pathogenic fungi by silver NPs through several mechanisms. -Significant inhibition of mycelium growth by increasing mycelium cell membrane permeability, inducing pathogen hypha shrinkage and distortion. -Decrease or complete inhibition of spore germination for all types of fungi, with reductions in two pathogens from 88.48% and 94.44% to 9.70% and 7.07%, respectively.	[29]
Antifungal	Green synthesis using *Trichoderma longibrachiatum*	-	-Significant inhibition of fungal pathogen, *Fusarium oxysporum* (*F. oxysporum*), by leading defects on the cell wall and structural damages. -Approximately 10% increase in both germination rate and germination potential of muskmelon. -Potential induction of reactive oxygen species (ROS) and cellular metabolism pathway interruption.	[30]
Antifungal	Green synthesized using *Cedrela odorata* (leaf and bark extracts)	Size (mean values) = 29.06 nm (leaf) and 19.80 nm (bark) SPR absorbance = 447.56 nm (leaf) and 439.44 nm (bark)	-Concentration-dependent fungicidal activity against *Fusarium circinatum*.	[31]
Antifungal	Green synthesized using *F. oxysporum*	Size = between 20 and 50 nm SPR absorbance = 415–420 nm Shape = spherical (The properties were taken from the methodology that researchers cited)	-Significant antifungal activity for onychomycosis treatment. -Mean MIC value 4.24 µg/mL and minimum fungicidal concentration from >3.31 to >42.50 µg/mL, depending on the fungal species. -Successful demonstration of antifungal activity ex vivo.	[32]
Food Packaging and Preservation	Green synthesized using *Ficus carica*	Size = ranging between 20 and 80 nm Shape = spherical	-Preservation of apple slices from browning and reduction in weight and moisture loss over time with biodegradable food packaging incorporating silver NPs. -Increase in thickness with the addition of NPs and alterations in water vapor permeability (WVP) and solubility. -High antioxidant capacity and antimicrobial activity due to the addition of silver NPs.	[33]
Food Packaging and Preservation	Green synthesis using grape seed extracts	Size = average size of 20 nm Shape = spherical	-Enhancement of shelf life of grapes by green synthesized silver NP and chitosan composite, preserving the texture and reducing weight loss over 5 days. -Antifungal activity and dose-dependent antioxidant capacity. -Significant reduction in total yeast and mold count on stored grapes.	[34]
Food Packaging and Preservation	NPs were synthesized using AgNO_3_ as the silver precursor and sodium citrate as a reducing agent, with cellulose nanocrystals serving as stabilizers	Size = average diameter of 10 to 20 nm Shape = spherical	-Enhanced shelf life of strawberries by silver NP coating onto a paper surface. -Significant antibacterial activity in the packaging structure, concentration-dependent. -Increased tensile strength by 1.26-fold, enhanced flexibility and ductility, decreased WVP by 45.4%, and reduced air permeability by 93.3%.	[35]
Food Packaging/Antifungal	Green synthesis using marine algae *Turbinaria turbinata*	Size = ranging from 14.50 to 39.85 nm Shape = spherical	-Significant antifungal activity. -Extended shelf-life, preserved quality, and delated microbiological decomposition of tomato samples for up to 17 days.	[36]
Nanofertilizer	Silver NPs were synthesized using feather hydrolysates obtained from the degradation of chicken feathers by *Bacillus safensis* (*B. safensis*) LAU 13 and *Aquamicrobium defluvii* (*A. defluvii*) FH 20	Size = average sizes of 42.01 ± 20.9 nm and 11.52 ± 6.37 nm for NPs synthesized from *B. safensis* and *A. defluvii*, respectively.	-At the highest concentration (150 µg/mL), silver NPs enhanced seed germination, shoot height, root length, leaf size, and chlorophyll content by up to 1.58-fold. -Increased radical scavenging capacity by 1.1-fold and inhibition of lipid peroxidation by up to 78% (1.21-fold). -Significant antifungal activity on three fungal strains, ranging from 60.33% to 88.2%.	[37]
Nanofertilizer	Silver NPs with a concentration of 200 ppm were purchased commercially	Size = around 40 to 60 nanometers Shape = spherical	-The highest concentration (15 mL/L) of spraying significantly enhanced the fruit’s physical characteristics: 48.9% increase in weight (grams), 38% in length (cm), and 44.4% in size (cm^3^). -Increased total sugar percentages by approximately 44%. -Insecticidal activity against various insects, with mortality rates ranging from 88.33% to 100% at 5000 ppm. -Increased pollen viability.	[38]
Nanofertilizer	Green synthesis using ascorbic acid, caffeic acid, and gallic acid	Size = 70 nm, between 50 and 80 nm, and 20 nm. Shape = spherical SPR absorbance = 440 nm, between 421 and 467 nm, and between 402 and 467 nm. (The properties were given respectively for gallic, caffeic, and ascorbic acid-synthesized particles.)	-Enhanced shoot and root lengths in cucumber seedlings. -Increased chlorophyll index and chlorophyll and carotenoid contents. -Variances in oxidative stress levels (both positive and negative).	[39]
Nano-pesticidal	Green synthesized using *Cassia fistula* (L.) leaf	Size = ranging from 10 to 20 nm Shape = spherical and oval	-Significant pesticidal activity against several tomato phytopathogens through a reduction in cell numbers and damage on the cell surface. -A significant reduction in biofilm formation. -Demonstration of antifungal activity against several fungal pathogens, evidenced by a 78% reduction in growth. -Inhibition of hatching (82% at 100 μg), galls formation (76%), and increased mortality (65.78% at 100 μg) of root-knot nematodes. -Increase in length (66%), lycopene (up to 52%), and dry biomass (58%) of tomatoes. -Increased levels of antioxidant enzymes by 47% and 60%.	[40]
Nano-pesticidal	Green synthesized using pecan nutshell extracts (*Carya illinoinensis*)	Size = approximate diameter of 50.2 nm Shape = hemispherical	-Strong insecticide activity with high mortality ratios, reaching up to 100% mortality with increased concentration and treatment time. -Under greenhouse conditions, the mortality ratio reached up to 80%.	[41]
Wound Healing	Green synthesized using green tea leaf extract, *Camellia sinensis* (*C. sinensis*)	Size = average diameter of 22.31 nm Shape = spherical	-Significant wound healing activity *in vitro* and *in vivo.* -Cell viability was above 70% in all tested samples. -The hydrogel significantly closed the wound gaps by 60–75% at day 8 and 98–99% at day 12.	[42]
Wound Healing	Silver NPs were synthesized using chemical reduction	Size = ranging between 10 and 20 nm Shape = spherical	-Significant antibacterial activity, biofilm removal (46.7% and 61.6%), and wound healing in infected burn wounds. -Reduction in the amount of blood loss by 4 times compared to the control group in a mouse liver trauma model (from 350 mg to 82 mg). -Noncompressive bleeding reduction in a rat liver defect model, from 1.04 g to 0.21 g. -A 96% total healed wound ratio after treatment, with the lowest TNF-α levels.	[43]
Wound Healing	-	-	-Accelerated healing effect of silver NPs (27.8 days) in administration on day 3 compared to other groups (35.8 and 40 days). -Inhibition of early inflammation by silver NP treatment at day 0. -Inhibition of prolonged inflammation in treatment on day 3.	[44]
Wound Healing	Chemical Synthesis	Size = predominantly between 27.25 and 49.61 nm Shape = spherical	-Silver NP-incorporated alginate gels demonstrated significant antibacterial activity and increased proliferation of HaCaT keratinocytes. -Suitable cytocompatibility and no induction of oxidative stress.	[45]
Wound Healing	Green synthesis through reduction of AgNO_3_ with lignin NPs and preparation with oxidation of cellulose nanofibrils	SPR absorbance = between 400 and 420 nm Shape = spherical	-Silver NP-included biofilm demonstrated significant UV protection (100% UVB and over 90% UVA) and antioxidant activity. -Enhanced mechanical properties, tensile strength, elongation at break, and WVP in optimum concentrations. -Antibacterial activity.	[46]
Bone Repair	-	-	-Osteogenesis and significant antibacterial activity by silver NP bone scaffolds. -A great increase in compressive strength and hardness and a reduction in water contact angle by approximately 17.5°. -Increased bone volume/total volume ratio by 55.7% at week 4 and 76.9% at week 8 in New Zealand rabbits.	[47]
Bone Repair	Green synthesized using *Trigonella foenum-graecum* extract	Size = average size of 118.0 ± 1.7 nm Shape = spherical	-Induction of osteocalcin levels between weeks 2 and 6. -Increase in calcium and phosphorus levels. -Near completion in bone repair progress, with mature bone formation at week 4.	[48]
Bone Repair	-	-	-Significant increase in proliferation of MC3T3-E1 cells between days 3 and 7 at a 200 μM concentration. -A significant increase in bone volume at concentrations of 200 μM and 400 μM.	[49]
Bone Repair	Chemically synthesized	Size = average 5–6 nm Shape = spherical	-Silver NP-containing antimicrobial bone scaffolds induced proliferation of osteoblasts.	[50]
Vaccine Adjuvant	Green synthesized using *F. oxysporum* 551 strain	Size = average 50 nm SPR absorbance = 420 nm Shape = spherical	-A vaccine combined with silver NPs against *Acinetobacter baumannii* (*A. baumannii*) protected mice against lethal infection. -Significant induction of IgG antibody response. -Prevented bacterial growth in lungs from mice.	[51]
Vaccine Adjuvant	Green synthesized using propolis extract		-Increased concentration (nearly doubled) of IL-4 with the addition of 30 mg/mL propolis silver NPs as adjuvants. -Concentration of IgG increased.	[52]
Vaccine Adjuvant	Purchased from NanoComposix, Europe	Size = 10 nm Shape= spherical	-Administration of silver NPs in the lungs of mice led to the recruitment of lymphoid cells (predominantly natural killer cells). -Enhancement of natural killer cell migration and IFN-γ production through macrophages.	[53]
Diabetes	Green synthesized using *Allium cepa*	Size = ranging between 49 and 73 nm Shape = spherical	-Significant *in vitro* antidiabetic activity by the inhibition of α-amylase and α-glucosidase by 70% and 55%, respectively. -Approximately 60% DPPH inhibition.	[54]
Diabetes	Green synthesized using *Psidium guajava* leaf extract	Size = ranging between 52.12 and 65.02 nm Shape = predominantly spherical	-A significant reduction in blood glucose levels, weight recovery, and restoration of lipid profiles to near control levels in streptozotocin-induced diabetic rats. -Improvements in liver and pancreatic cells in histopathological analysis.	[55]
Diabetes	Green synthesized using *Allium sativum*	Size = ranging from 10 to 30 nm Shape = spherical	-Increase in glucose uptake ranging from 28.9% to 41.54%. -Inhibition of glucose production by 26.28% to 57.74%. -Antioxidant activity ranging from 31% to 63%, dose-dependently (20 to 100 µg/mL). -A significant inhibition of α-amylase and α-glucosidase enzymes through the interaction of silver atoms with amino acid residues.	[56]
Diabetes/Wound Healing	Green synthesized using *Cyanobacteria Synechocystis* sp.	Size = diameters ranging from 10 to 35 nm Shape = spherical	-Diabetic wound healing by 89.4% and wound closure by 50.96% in diabetic rats. -Complete wound restoration with treatment on day 21 with increased levels of angiogenesis-related factors.	[57]
Diabetes/Wound Healing	Chemical Synthesis	Size = 2–12 nm SPR absorbance = 420–430 nm Shape = spherical	-Compatible wound dressing characteristics. -High cytocompatibility and hemocompatibility for diabetes-induced wound dressing. -Significant antibacterial activity.	[58]
Diabetes	Green synthesis using *Salvia blepharophylla* (*S. blepharophylla*) and *Salvia greggii* (*S. greggii*)leaf extracts	Size = average of 52.4 nm (*Salvia blepharophylla*) and 62.5 nm (*Salvia greggii)* Shape = spherical	-Significant antidiabetic activity through α-amylase inhibition of up to 86.5%, depending on the concentration. -Antioxidant and antibacterial activity also demonstrated.	[59]
Diabetes	Green synthesis using *Azadirachta indica* seed extract	Size = average 34.43 nm SPR absorbance = between 400 and 450 nm Shape = spherical	-Silver NPs demonstrated α-amylase inhibition by 73.85%, glucose absorption by 10.65%, and glucose uptake by yeast cells of 75%. -Significant inhibition of blood glucose levels of mice (420 to 290 mg/dL at the highest concentrations).	[60]
Diabetes	Green synthesis using *Cucumis melo* L. leaf extract	Size = between 66.7 and 92.3 nm Shape = spherical	-Significant α-amylase and α-glucosidase inhibitory activity by 65.6% and 63.1% at the highest concentration (100 μg/mL), respectively. -Anticoccidial activity.	[61]
Dental (Oral Disease)	Silver NPs were synthesized through chemical reduction, using AgNO_3_ with gallic acid as a reducing and stabilizing agent	Size = two different sizes, 5.2 ± 1.2 and 37.4 ± 3.6 nm Shape = spherical and semispherical, respectively	-Significant antimicrobial activity against biofilms from patients with dental caries.	[62]
Dental (Acrylic Resin)	Silver NPs were synthesized through AgNO_3_ reduction by sodium citrate	Size = 5 and 10 nm Shape = spherical	-Biofilm inhibition on acrylic resin with silver NP nanocomposite incorporation. -No alterations in inflammatory responses and flexural strength of acrylic resin.	[63]
Dental (Acrylic Resin)	Chemical synthesis	Size = 25 nm	-Resistance to bacterial adhesion. -Significant bactericidal activity. -Stabile and self-cleaning coating.	[64]
Dental (Composite Resin)	Silver NPs were synthesized through chemical reduction, using AgNO_3_ with sodium borohydride as a reducing agent	Size = 33.5 nm Shape = spherical	-Increased antibacterial activity and enhancement of compressive strength in silver NP-containing resin composite.	[65]
Dental (Composite Resin)	Chemical synthesis	Size = average 26.5 nm SPR absorbance = 407 nm Shape = spherical	-Significant antibacterial activity of silver NP-containing composite resin against multiple strains.	[66]
Dental (Periodontal Restoration)	Silver NPs were synthesized through chemical reduction, using ascorbic acid as a reducing agent and sodium citrate as a stabilizing agent	Size = average diameter of 30 nm Shape = spherical	-Increased tensile strength and decreased elongation at break obtained with collagen–silver NP hydrogels. -Significant antibacterial activity, high inhibition zone (concentration-dependent), and slight increase in proliferation of human gingival fibroblast.	[67]
Dental (Periodontal Restoration)	Chemical synthesis	Size = 10.2 and 29.3 nm Shape = spherical	-Significant bactericidal and antibiofilm activity of silver NPs against oral biofilms from patients.	[68]
Dental (Root Canal Filming)	Chemical synthesis	Size = 5.57 nm Shape = spherical	-No influence on mechanical properties, bonding strength, or surface roughness.	[69]
Dental (Orthodontics)	Chemical synthesis	-	-Lowered bacterial adherence with silver NP-coated orthodontic brackets.	[70]
Dental (Orthodontics)	Silver NPs were synthesized through chemical reduction, using Augmentin as both a reducing agent and a coating material to stabilize the particles	Size = ranging in diameter from 50 to 80 nm Shape = spherical	-Antibacterial activity and enhanced shear bond strength by augmentin-coated silver NPs on orthodontic cement without any toxicity.	[71]
Dental (Implant)	Silver NPs were synthesized through *in situ* reduction, using dopamine as the reducing agent	Size = ranging from 20 to 30 nm	-Significant antibacterial activity with prevention of bacterial adhesion and colonization.	[72]
Anticancer	Green synthesized using Actinobacterial strain SF23	Size = mean of 13.2 nm Shape = spherical	-Significant cytotoxic activity on MCF-7 cancer cells and RAW 264.7 macrophages. -With increased ROS levels, cell viabilities were reduced to 15.8% and 14.2%, respectively.	[73]
Anticancer	Green synthesized using the *Dictyota ciliolata* extract	Size = average size of 100 nm Shape = spherical	-Anticancer activity against lung adenocarcinoma A549 cells through ROS induction and DNA damage, leading to morphological changes. -Expressions of Caspase-3, Bcl2, and Bax induced.	[74]
Anticancer	Green synthesized using *Swietenia macrophylla* seed extract	Size = ranging between 10 and 23 nm Shape = spherical or oval	-Significant anticancer activity through oxidative stress. -High DNA damage from S-phase cycle arrest (20.3% increase) and decrease in G1/G0 phase (77.7%).	[75]
Anticancer	Green synthesized using *Cissus woodrowii* leaf extract	Size = ranging between 20 and 30 nm Shape = spherical	-Significant inhibition of cell proliferation in breast cancer cells. -Induction of apoptosis through up-regulated expression of both p53 and caspase-3 genes and down-regulation of Bcl2 (both protein and mRNA levels).	[76]
Anticancer	Green synthesized using extracellular filtrate of *F. oxysporum*	Size = ranging from 6.53 to 21.84 nm Shape = spherical	- In vitro anticancer activity against HepG2 and MCF-7 cells. - Half-maximal inhibitory concentration (IC_50_) values determined as 7.6 µg/mL and 35.4 µg/mL, respectively. -Interaction with FGF19 and BCL-2 proteins.	[77]
Anticancer	Green synthesis using tomato flower waste extracts	Size = ranging from 14 to 40 nm Shape = predominantly spherical SPR absorbance = ranging between 400 and 500 nm	-Significant antitumor potential against HeLa and HT29 cell lines. -Cell viability reductions of 50.49% and 62.45%. -Observable cell deformation.	[78]
Bioimaging/Biosensor	Green synthesis using black tea extract (*C. sinensis*)	Size = 52.3 nm Shape = spherical	-A novel electrochemical sensor based on incorporating silver NPs and carbon black on chitosan films was developed. -Close to 100% recovery rate was recorded in the analysis of ciprofloxacin in synthetic urine samples.	[79]
Bioimaging/Biosensor	Silver NPs were synthesized through the laser ablation method	Size = 26 nm Shape = spherical	-LSPR-active silver NPs were developed to detect trenbolone acetate dopants, which is crucial in anti-doping efforts in sports. -A 9.12 ppb limit of detection was achieved, meeting World Anti-Doping Agency (WADA) standards.	[80]
Bioimaging/Biosensor	Silver NPs were synthesized through chemical reduction, using sodium citrate as a reducing agent	Size = approximately 70 ± 20 nm Shape = spherical	-A novel, highly sensitive surface-enhanced Raman scattering (SERS) chip based on silver NPs nanocomposites was developed. -Efficient detection of DNA bases (adenine) with a limit of detection of 0.026 pM.	[81]
Bioimaging/Biosensor	Silver NPs were synthesized through a photochemical reduction method, using ultraviolet-C (UVC) light as the reducing agent	-	-Development of Mn_2_O_3_–silver nanocomposites for the sensitive detection of Nitrofurazone, an antibiotic linked to potential abnormalities in human embryos or fetuses. -Achievement of an ultralow limit of detection, 7.39 × 10^−13^ M, along with an enhancement factor of 2.05 × 10^12^ in SERS performance.	[82]
Bioimaging/Biosensor	Synthesis by the Tollens method	Plasmon resonance band = around 455 nm Size = mostly distributed around 75 and 95 nm Shape = spherical and semi-spherical	-Detection of *Shigella* bacteria by quick and sensitive silver NP-based SERS system. -High enhancement factor and low limits of detection. -Antibacterial activity, mediated by silver ions, against *Shigella* bacteria.	[83]
Bioimaging/Biosensor	Chemical synthesis	Size = between 16 and 25 nm SPR absorbance = 317 nm	-Sensitive and strong detection of the chikungunya virus. -The sensor demonstrated a limit of detection of 0.1 ng/mL and a recovery rate between 91% and 93%.	[84]

## Data Availability

No new data were created or analyzed in this study.
